# Mechanisms of APOBEC3 mutagenesis in human cancer cells

**DOI:** 10.1038/s41586-022-04972-y

**Published:** 2022-07-20

**Authors:** Mia Petljak, Alexandra Dananberg, Kevan Chu, Erik N. Bergstrom, Josefine Striepen, Patrick von Morgen, Yanyang Chen, Hina Shah, Julian E. Sale, Ludmil B. Alexandrov, Michael R. Stratton, John Maciejowski

**Affiliations:** 1 Broad Institute of MIT and Harvard, Cambridge, MA USA; 2 Molecular Biology Program, Sloan Kettering Institute, Memorial Sloan Kettering Cancer Center, New York, NY USA; 3 Department of Cellular and Molecular Medicine, UC San Diego, La Jolla, CA USA; 4 Department of Bioengineering, UC San Diego, La Jolla, CA USA; 5 Moores Cancer Center, UC San Diego, La Jolla, CA USA; 6 Division of Protein & Nucleic Acid Chemistry, Medical Research Council Laboratory of Molecular Biology, Cambridge, UK; 7 Cancer, Ageing and Somatic Mutation, Wellcome Sanger Institute, Hinxton, UK

**Keywords:** Cancer genomics, Genomic instability

## Abstract

The APOBEC3 family of cytosine deaminases has been implicated in some of the most prevalent mutational signatures in cancer^1–3^. However, a causal link between endogenous APOBEC3 enzymes and mutational signatures in human cancer genomes has not been established, leaving the mechanisms of APOBEC3 mutagenesis poorly understood. Here, to investigate the mechanisms of APOBEC3 mutagenesis, we deleted implicated genes from human cancer cell lines that naturally generate APOBEC3-associated mutational signatures over time^4^. Analysis of non-clustered and clustered signatures across whole-genome sequences from 251 breast, bladder and lymphoma cancer cell line clones revealed that *APOBEC3A* deletion diminished APOBEC3-associated mutational signatures. Deletion of both *APOBEC3A* and *APOBEC3B* further decreased APOBEC3 mutation burdens, without eliminating them. Deletion of *APOBEC3B* increased APOBEC3A protein levels, activity and APOBEC3A-mediated mutagenesis in some cell lines. The uracil glycosylase UNG was required for APOBEC3-mediated transversions, whereas the loss of the translesion polymerase REV1 decreased overall mutation burdens. Together, these data represent direct evidence that endogenous APOBEC3 deaminases generate prevalent mutational signatures in human cancer cells. Our results identify APOBEC3A as the main driver of these mutations, indicate that APOBEC3B can restrain APOBEC3A-dependent mutagenesis while contributing its own smaller mutation burdens and dissect mechanisms that translate APOBEC3 activities into distinct mutational signatures.

## Abstract (web summary)

Endogenous APOBEC3 deaminases generate prevalent mutational signatures in human cancer cells, and APOBEC3A is the main driver of these mutations.

## Main

Early investigations into the patterns of mutations in cancer genomes revealed that cytosine mutations are commonly present in TCN (where N is any nucleotide) trinucleotide sequence contexts^1,2,5^. The sequence context preferences of the APOBEC cytosine deaminases, which target DNA and RNA of viruses and retroelements as part of the innate immune defence, led to the proposal that such mutations derive from APOBEC activity^1–3,6,7^. Mathematical deconvolution of patterns of single-base substitutions (SBSs) from cancer genomes uncovered different mutational signatures of non-clustered (termed signatures SBS2 and SBS13) and clustered (kataegis and omikli) APOBEC-associated cytosine mutations at TCN trinucleotides^1,8,9^. APOBEC-associated mutational signatures have been identified in more than 70% of cancer types and around 50% of all cancer genomes, with prominence in breast and bladder cancer as well as other cancer types^10,11^. Indirect links implicate the APOBEC3 family as a source of these mutations: (1) *APOBEC3* overexpression in model systems produces cytosine mutations with features that are similar to SBS2 and SBS13 and can contribute to carcinogenesis; (2) polymorphisms at the *APOBEC3* locus are, in some contexts, associated with cancer mutations, and (3) APOBEC3 activity in cancer has been inferred using surrogate measures, including expression, in vitro deamination and RNA editing of model substrates^3,12,13^.

However, causal links between endogenous APOBEC3s and mutational signatures in human cancer genomes have not been established, leaving the impacts of individual enzymes poorly understood^3,13^. Among candidate APOBEC3 enzymes, expression of *APOBEC3B* is the highest in cancer and moderately correlates with APOBEC3-associated mutational burdens^14,15^. *APOBEC3B* expression is associated with worse clinical outcomes in oestrogen-receptor-positive breast cancer^16,17^. APOBEC3B was reported to be the only enzyme with detectable DNA deaminase activity in cell extracts from >75% of breast cancer cell lines^14^. On the basis of these and other observations drawn from expression and deamination-based assays, APOBEC3B is often considered to be a major mutator and therapeutic target in breast and other cancer types^14,15,18–20^. However, an *APOBEC3B* germline deletion polymorphism is associated with increased cancer risk and higher APOBEC3-associated mutation burdens in certain contexts, suggesting mutator roles for additional APOBEC3s^3,21,22^. Indeed, other links suggest a more prominent role for APOBEC3A. APOBEC3-associated mutations in cancers more frequently present in APOBEC3A-preferred YTCA sequence contexts, compared with APOBEC3B-preferred RTCA sequence contexts (where Y indicates a pyrimidine base and R is a purine base, and C is the mutated base)^23^. Furthermore, APOBEC3A was recently reported to have stronger deamination activity in breast cancer cell lines under certain conditions and to be the better correlate with mutation load in breast tumours compared with APOBEC3B^24^; to promote tumorigenesis in mouse models predisposed to cancer after overexpression^25^; and to contribute to recurrent mutations at DNA hairpins in cancer^26,27^.

It is critical to establish the relative contributions of individual, endogenous APOBEC3 enzymes to mutation burdens in human cancer genomes to understand the aetiology of major mutation burdens in cancer and to enable correctly focused investigations of widely discussed APOBEC3-focused therapeutic strategies^18,19^. Progress in testing the mutagenic capacity of individual APOBEC3 enzymes in the endogenous setting has been hindered by differences between the human and mouse *APOBEC3* loci and the lack of characterized human cancer cell models with endogenous APOBEC3 mutagenesis. To resolve these debates, we used a workflow to directly measure mutagenesis by individual, endogenous APOBEC3 enzymes in human cancer cells.

## APOBEC mutagenesis in cancer cells

**Fig. 1:**
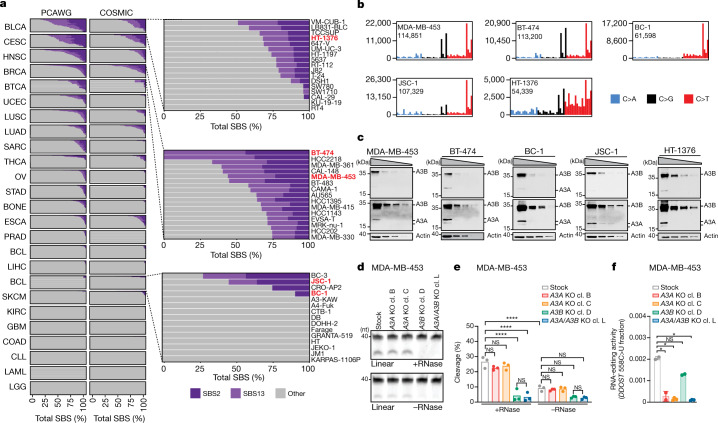
Human cancer cell line models of APOBEC3-associated mutagenesis. **a**, The prevalence of the SBS2 and SBS13 signatures in 1,843 whole-genome-sequenced human cancers and 780 whole-exome-sequenced COSMIC cancer cell lines. Each bar represents the percentage of mutations attributed to the indicated SBS signatures in an individual sample from the indicated cancer types. Abbreviations are defined in Supplementary Table 1. Subsets of the BLCA, BRCA and BCL datasets are magnified to highlight the cell lines chosen for further study (red). **b**, The mutational profiles of the indicated cell lines plotted as the numbers of genome-wide substitutions (*y* axis) at cytosine bases classified into 48 possible trinucleotide sequence contexts (*x* axis; Extended Data Fig. 4a). **c**, Immunoblotting with anti-APOBEC3 (04A04) and anti-actin antibodies. Extracts (40 µg, 20 µg, 10 µg and 5 µg) were prepared from the indicated cell lines. The anti-APOBEC3 antibody detects APOBEC3A and APOBEC3B (Extended Data Fig. 1g). Multiple exposures are shown to better depict APOBEC3A (A3A) and APOBEC3B (A3B) signals. *n* = 3 experiments. **d**, Cytosine deaminase activity in the indicated cell lines was measured against a linear probe with (top) or without (bottom) RNase treatment to degrade RNA in the extracts. cl., clone; nt, nucleotides. **e**, Quantification of APOBEC3 deaminase activity as the percentage of processed DNA as in **d**. Data are mean. Statistical analysis was performed using one-way analysis of variance (ANOVA) with Tukey multiple-comparisons test; ****P *<* 0.0001; NS, not significant. *n* = 3 experiments. **f**, Quantification of *DDOST* 558C>U levels in the indicated MDA-MB-453 cells. Data are mean ± s.d. Statistical analysis was performed using one-way ANOVA with Tukey multiple-comparisons test; *P < 0.05; NS, not significant. *n* = 2 experiments.

To assess whether cell lines are suitable models of endogenous APOBEC3 mutagenesis, we compared APOBEC3-associated mutational signatures across DNA sequences of 780 human cancer cell lines^4^ and 1,843 cancers^4,10^ (Fig. 1a and Supplementary Table 1). The prevalence of SBS2 and SBS13 in cell lines closely resembled their prevalence across matching cancer types. For example, whereas cancers of breast, bladder, head and neck, and cervix are among the most affected, colorectal and kidney cancers rarely present with the relevant signatures. These similarities suggest that the presence of APOBEC3-associated signatures in cell lines reflect traces of processes with in vivo origins rather than mutational processes associated with in vitro cultivation.

**Extended Data Fig. 1:**
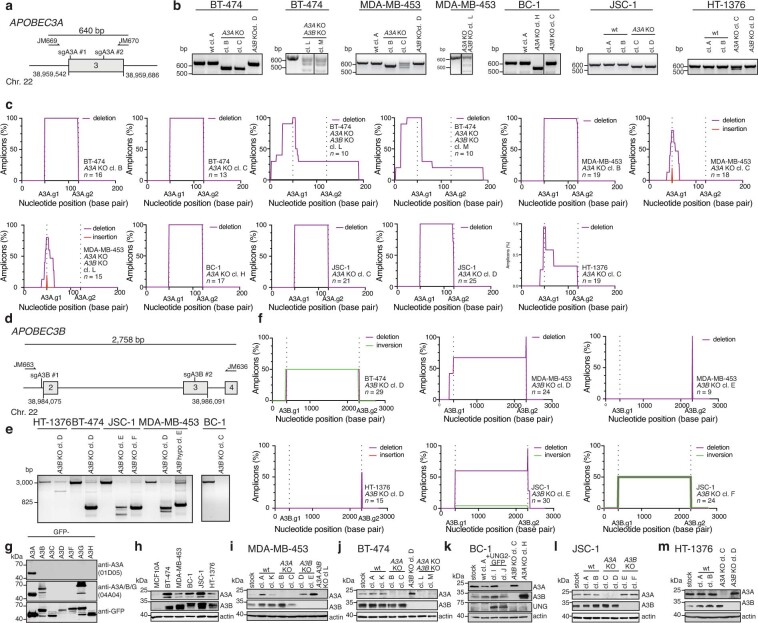
Generation of *APOBEC3A* and *APOBEC3B* knockout cell line clones. **a)** Schematic of *APOBEC3A* locus. Position of exon 3, targeting sgRNAs (sgA3A #1 and sgA3A #2) and primers for PCR screening (JM669 and JM670) are indicated. **b)** PCR amplicons generated using primers JM669 and JM670 and genomic DNA templates prepared from the indicated cell lines. *n* = 2 experiments. **c)** Plots depict a percentage of sequenced amplicons generated as in **b** that contain deletions (purple) or insertions (red) at the indicated positions. **d)** Schematic of the *APOBEC3B* locus. Position of exons 2–4, targeting sgRNAs (sgA3B #1 and sgA3B #2) and primers for PCR screening (JM663 and JM636) are indicated. **e)** PCR amplicons generated using primers JM663 and JM636 and genomic DNA templates prepared from the indicated cell lines. *n* = 2 experiments. **f)** Plots depict a percentage of sequenced amplicons generated as in **e** that contain deletions (purple) and inversions (green). **g)** Immunoblotting with anti-APOBEC3A (01D05), anti-APOBEC3A/B/G (04A04), and anti-GFP antibodies in extracts prepared from HEK293FT cells transfected with the indicated GFP-APOBEC3 constructs. *n* = 3 experiments. **h-m)** Immunoblotting with anti-APOBEC3A (01D05), anti-APOBEC3 (04A04), anti-UNG, and anti-actin antibodies in the indicated cell lines (h-j,l,m, 3 experiments; k, *n* = 2 experiments).

**Extended Data Fig. 2:**
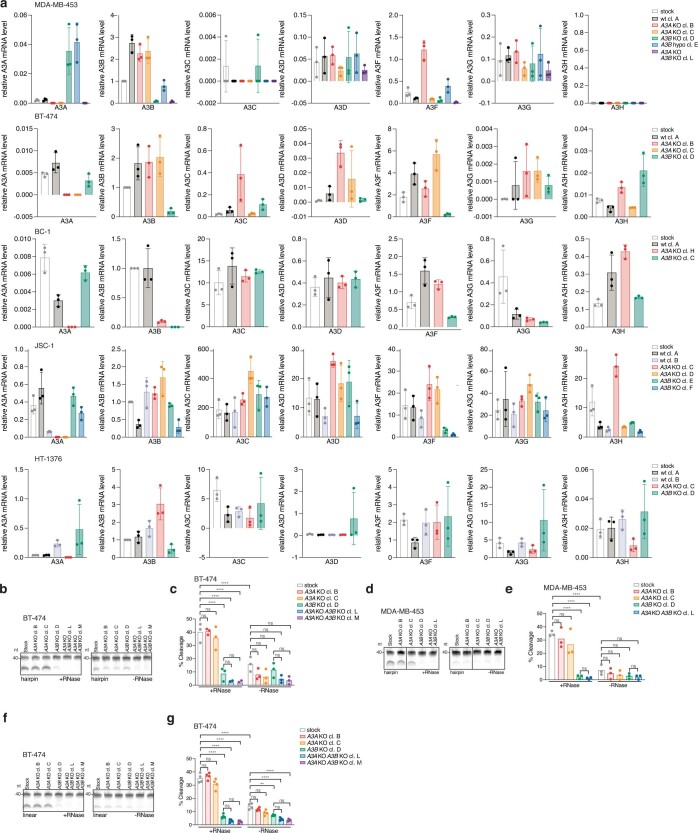
APOBEC3 expression and deaminase activity in cancer cell lines. **a)** Normalized APOBEC3 mRNA levels in the indicated cell lines based on qPCR. The mean ± S.D. of *n* = 3 independent biological replicates are shown. **b**,**d**,**f)** Cytosine deaminase activity in the indicated cell lines measured against linear or hairpin probes ± RNase treatment to degrade RNA in extracts. **c**,**e**,**g)** Quantification of APOBEC3 deaminase activity as a percentage of processed DNA as in b,d,f) (Mean, ****p < 0.0001; **p < 0.01 ns, not significant, one-way ANOVA with Tukey’s multiple-comparisons test, c,e) *n* = 3, g) *n* = 4).

To investigate mechanisms of endogenous APOBEC3 mutagenesis, we deleted *APOBEC3A* and *APOBEC3B* from a panel of cancer cell lines that acquire APOBEC3-associated mutations over time^4^ (Extended Data Fig. 1). The panel included breast cancer (BRCA; BT-474, MDA-MB-453), B cell lymphoma (BCL; BC-1, JSC-1) and bladder cancer (BLCA; HT-1376) cell lines (Fig. 1b). We next used surrogate assays of APOBEC3 mutagenesis across stock cell lines and *APOBEC3A-* and *APOBEC3B*-knockout clones to assess the relative roles of candidate APOBEC3 mutators in generating APOBEC3-associated mutations. Consistent with the widely reported observations of upregulation of *APOBEC3B* in breast and other cancer types^14,15^, all of the cell lines exhibited substantially elevated mRNA and protein levels of APOBEC3B relative to APOBEC3A (Fig. 1c and Extended Data Fig. 2a). Analyses across individual clones revealed that APOBEC3A and APOBEC3B expression varied, but APOBEC3B was uniformly more abundant than the minimally expressed APOBEC3A (Extended Data Fig. 2a). Consistent with its elevated expression levels, APOBEC3B was the major source of cytosine deaminase activity against both linear and hairpin probes in MDA-MB-453 and BT-474 extracts (Fig. 1d,e and Extended Data Fig. 2b–g).

Although we could not detect statistically significant decreases in cytosine deaminase activity in *APOBEC3A*-knockout cell extracts under any of the conditions tested, we could detect weak APOBEC3A-derived activity that seemed to be stronger than APOBEC3B under some conditions (hairpin substrates; cellular RNA present), in agreement with a previous report^24^ (Extended Data Fig. 2b–e). We could also measure low, APOBEC3A-associated RNA editing activity against a model hotspot located within *DDOST* transcripts in MDA-MB-453 cells using a droplet digital PCR assay^28^ (Fig. 1f). Analysis of cytosine mutations in APOBEC3A-preferred YTCA and APOBEC3B-preferred RTCA sequence contexts^23^ revealed enrichment of cytosine mutations in APOBEC3A-preferred contexts in MDA-MB-453, BT-474, BC-1 and JSC-1 cells^4^. Thus, high expression levels and deaminase activity seemingly implicate APOBEC3B as the major mutator in all of the cancer cell lines analysed here, whereas analyses of extended sequence contexts and RNA-editing assays suggest a potential role for APOBEC3A. These data recapitulate widely reported findings about the activities of APOBEC3A and APOBEC3B that produced the ongoing debate regarding the relevance of each enzyme in causing mutations in cancer^3,12,13^.

**Fig. 2:**
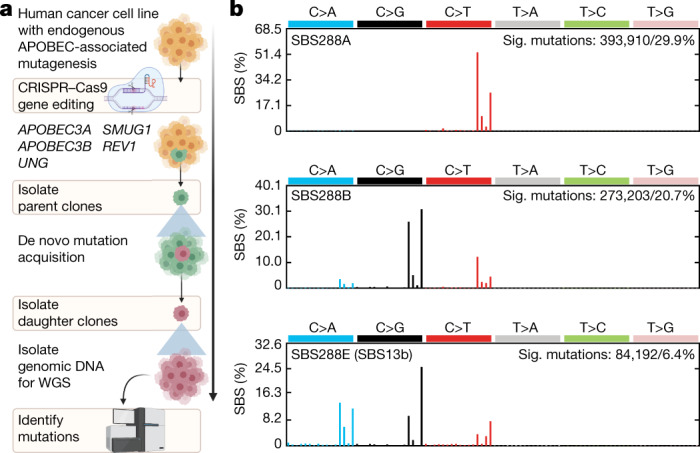
Using human cancer cell lines to investigate the origins of APOBEC3-associated mutagenesis. **a**, The experimental design used to track mutation acquisition in vitro over specific timeframes. The schematic was generated using BioRender. **b**, Profiles of APOBEC-associated signatures (sig.) extracted from SBSs identified across mutational catalogues of 5 stock cell lines and 251 parent and daughter clones. Mutational profiles are plotted as the percentage of genome-wide substitutions (*y*-axis) at cytosine or thymine bases classified into 96 possible trinucleotide sequence contexts (*x*-axis; Extended Data Fig. 4a). Subsequent deconvolution into COSMIC signatures revealed that SBS288A corresponds to COSMIC reference signature SBS2, SBS288B to SBS13 (termed SBS13a), whereas SBS288E represents a new version of COSMIC SBS13, which was termed SBS13b and quantified across samples in its extracted (rather than COSMIC) form. PCAWG, pan-cancer analysis of whole genomes; WGS, whole-genome sequencing.

**Extended Data Fig. 3:**
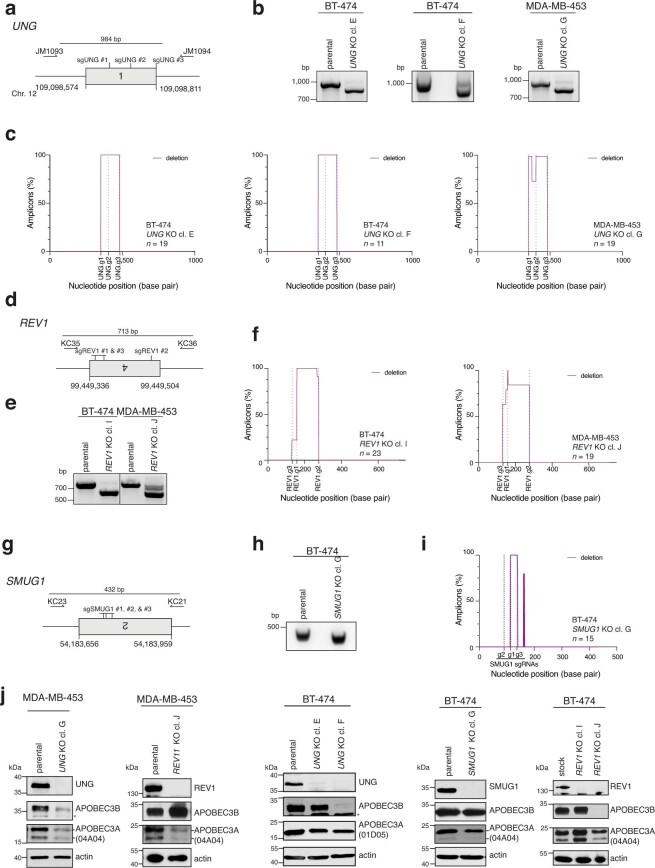
Generation of *REV1* and *UNG* knockout cell line clones. **a)** Schematic of *UNG* locus. Position of exon 1, targeting sgRNAs (sgUNG #1, sgUNG #2 and sgUNG#3) and primers for PCR screening (JM1093 and JM1094) are indicated. **b)** PCR amplicons generated using primers JM1093 and JM1094 and genomic DNA templates prepared from the indicated cell lines. *n* = 2 experiments. **c)** Plots depict a percentage of sequenced amplicons generated as in **b** that contain deletions (purple). **d)** Schematic of *REV1* locus. Position of exon 4, targeting sgRNAs (sgREV1 #1, sgREV1 #2 and sgREV1 #3), and primers for PCR screening (KC35 and KC36) are indicated. **e)** PCR amplicons generated using primers KC35 and KC36 and genomic DNA templates prepared from the indicated cell lines. *n* = 2 experiments. **f)** Plots depict a percentage of sequenced amplicons generated as in **e** that contain deletions (purple). **g)** Schematic of *SMUG1* locus. Position of exon 2, targeting sgRNAs (sgSMUG1 #1, sgSMUG #2 and sgSMUG #3), and primers for PCR screening (KC23 and KC21) are indicated. **h)** PCR amplicons generated using primers KC23 and KC21 and genomic DNA templates prepared from the indicated cell lines. *n* = 2 experiments. **i)** Plots depict a percentage of sequenced amplicons generated as in **h** that contain deletions (purple). **j)** Immunoblotting with anti-APOBEC3A (01D05), anti-APOBEC3 (04A04), anti-UNG, anti-REV1, anti-SMUG1, and anti-actin antibodies in the indicated cell lines. *n* = 3 experiments.

To resolve these discrepancies, we directly monitored acquisition of APOBEC3-associated mutations in cancer cell lines over time^4^ (Fig. 2a and Extended Data Figs. 1 and 3). Single-cell derived wild-type or knockout parent clones were subjected to long-term cultivation over 60–143 days, corresponding to a timeframe over which mutation acquisition was investigated. After this period, a further round of subcloning was carried out on the cell population from each of these parent clones. Multiple single-cell derived daughter clones were expanded into a population of cells for DNA isolation. In total, 251 individual parent and daughter clones were obtained and analysed using whole-genome sequencing (Supplementary Table 1). The workflow enabled the detection of mutations that were absent in parent clones, but present in the corresponding daughter progeny, therefore identifying mutations that were acquired over defined periods of in vitro propagation across different genetic backgrounds (Methods).

**Extended Data Fig. 4:**
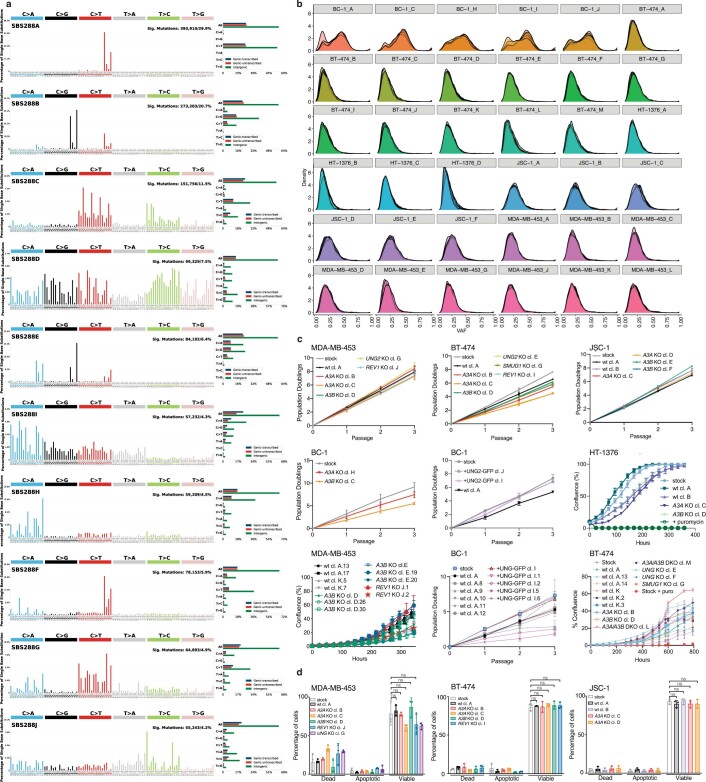
Mutational signature extraction and cancer cell line characterization. **a)** Profiles of mutational signatures de novo extracted from SBS identified in mutational catalogues from 5 stock cell lines and 251 parent and daughter clones. Each signature is displayed as the percentage of mutations (y-axis) attributed to 96 SBS classes (x-axis), which are defined by the colour-coded substitution class and sequence context immediately 3′ and 5′ to the mutated base. **b)** Distributions of variant allele fractions (VAFs) of mutations identified in daughter clones from the parental lineages indicated on top. **c)** Population doubling measures over successive passages or confluency measurements from the indicated cell lines. Mean ± SD from *n* = 3 independent biological replicates are shown. **d)** Plots showing percentages of apoptotic, necrotic, or living cells as indicated by propidium iodide and annexin V staining (Mean ± SD, ns, not significant, one-way ANOVA with Tukey’s multiple-comparisons test, *n* = 3).

To deconvolute APOBEC3-associated mutations from mutations induced by other processes, mutational signatures were extracted from mutational catalogues generated from whole-genome sequences of 251 clones and 5 bulk cell lines (Methods and Supplementary Tables 2 and 3). Ten de novo signatures were identified, three of which were characterized by APOBEC3-associated mutations in TCN contexts (SBS288A; similar to SBS2^10^ characterized by C>T mutations; SBS288B, similar to SBS13^10^ characterized by C>G and C>A mutations; and SBS288E, similarto SBS13 albeit with a higher relative proportion of C>A mutations; Fig. 2b, Extended Data Fig. 4a and Supplementary Table 4). Discovered signatures were decomposed into COSMIC reference signatures, yielding a final set of signatures that were subsequently quantified across individual samples (Methods and Supplementary Table 4). These included APOBEC3-associated SBS2; SBS13a (corresponding to COSMIC SBS13); SBS13b (corresponding to SBS288E); SBS1 and SBS5, signatures of processes that operate continuously across most normal and cancer cells^29,30^; SBS18, characterized by C>A mutations, in part attributed to reactive oxygen species^10^; new signatures of probable in vitro processes, which were not identified in cancer before and which presented across multiple lineages of mostly individual cell lines (SBS288D in HT-1376, SBS288I in BT-474 and SBS288J in MDA-MB-453); and other known signatures (SBS10b, SBS30, SBS38) that contributed small mutation burdens and probably represent false-positive attributions (Methods). For simplicity, mutation burdens of all but APOBEC3-associated signatures were grouped into the class ‘SBS other’.

## APOBEC3A in SBS2 and SBS13 generation

**Fig. 3:**
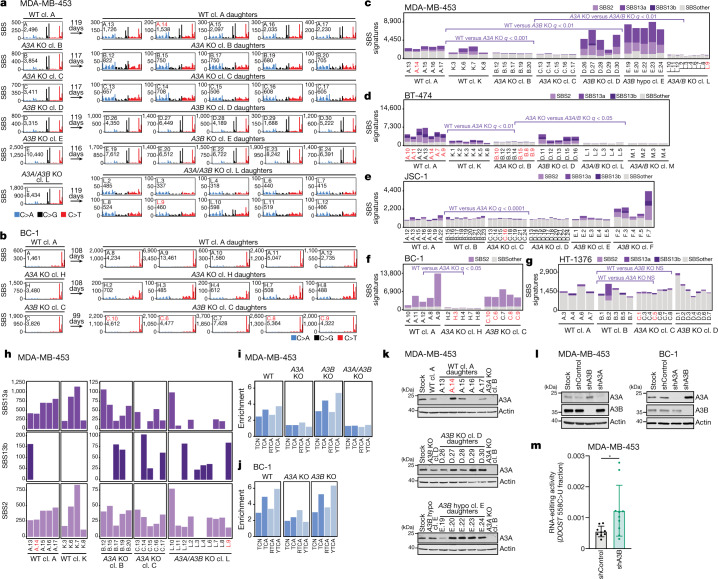
APOBEC3 deaminases drive the acquisition of SBS2 and SBS13 in human cancer cells. **a**,**b**, Mutational profiles of the indicated MDA-MB-453 (**a**) and BC-1 (**b**) clones plotted as the numbers of genome-wide substitutions (*y* axis) at cytosine bases classified into 48 possible trinucleotide sequence contexts (*x* axis; Extended Data Fig. 4a). cl., clone. The arrows indicate the number of days spanning the cloning events of parents (left of arrow) and daughters (right) during which mutation acquisition was tracked. **c**–**g**, The numbers of SBSs attributed to colour-coded mutational signatures discovered in the indicated daughter clones from the MDA-MB-453 (**c**), BT-474 (**d**), JSC-1 (**e**), BC-1 (**f**) and HT-1376 (**g**) cell lines with the indicated genotypes. *q* values comparing cumulative counts of SBS2, SBS13a, and SBS13b were calculated using one-tailed Mann–Whitney *U-*tests and false-discovery rate (FDR)-corrected using the Benjamini–Hochberg procedure. hypo, hypomorph. **h**, Focused plots showing SBS2 and SBS13a/b burdens in the indicated daughter clones. **i**,**j**, Enrichment of cytosine mutations in APOBEC3B-preferred RTCA and APOBEC3A-preferred YTCA sequence contexts in the indicated MDA-MB-453 (**i**) and BC-1 (**j**) daughter clones. R, purine base; Y, pyrimidine base; N, any base. **k**,**l**, Immunoblotting using anti-APOBEC3A (01D05), anti-APOBEC3B and anti-actin antibodies in the indicated cell lines. **m**, Quantification of *DDOST* 558C>U levels in the indicated MDA-MB-453 cells. Data are mean ± s.d. Statistical analysis was performed using two-tailed Student’s *t*-tests; *P < 0.05. *n* = 9 experiments. Clones marked in red font were excluded from statistical tests (Methods). Data from additional cell lines are shown in Extended Data Figs. 6 and 7.

**Extended Data Fig. 5:**
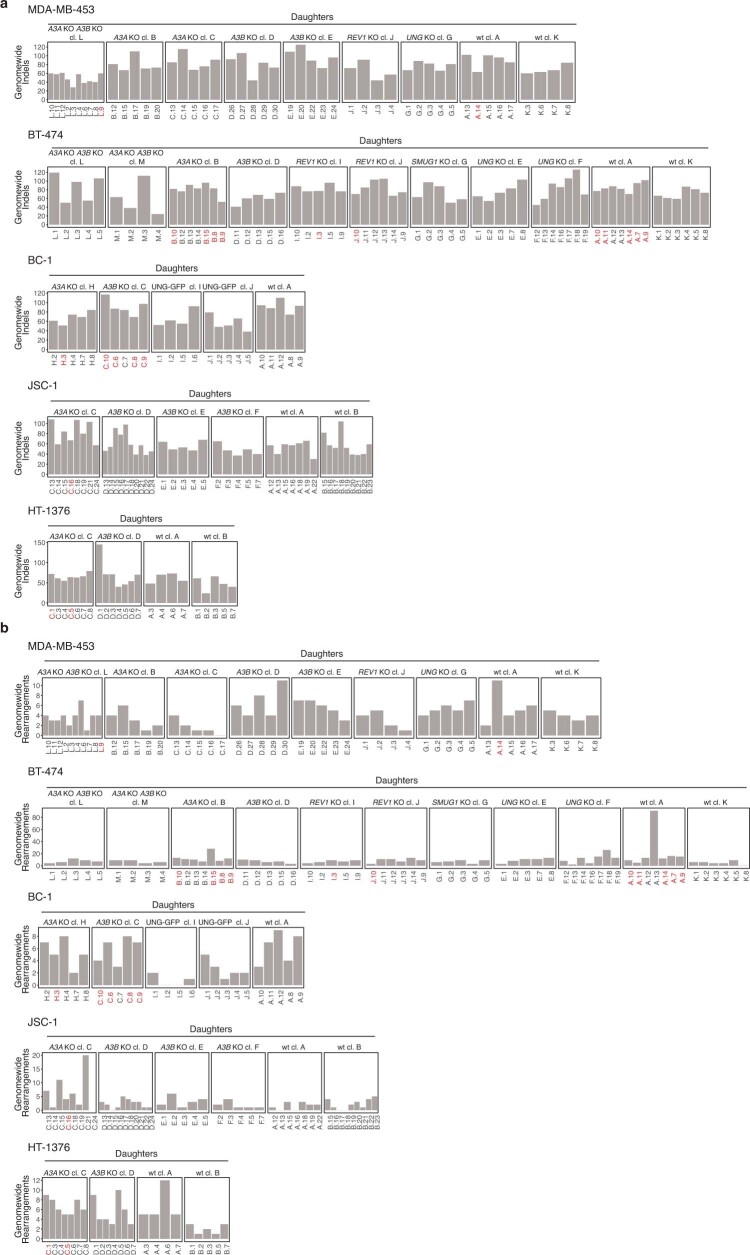
Analysis of indels and chromosome rearrangements across cell line clones. **a)** Plots showing numbers of indels and **b)** chromosome rearrangements detected genome-wide in the indicated cell line clones. Clones marked in red were excluded from statistical tests on mutational burdens across the study (Methods).

**Extended Data Fig. 6:**
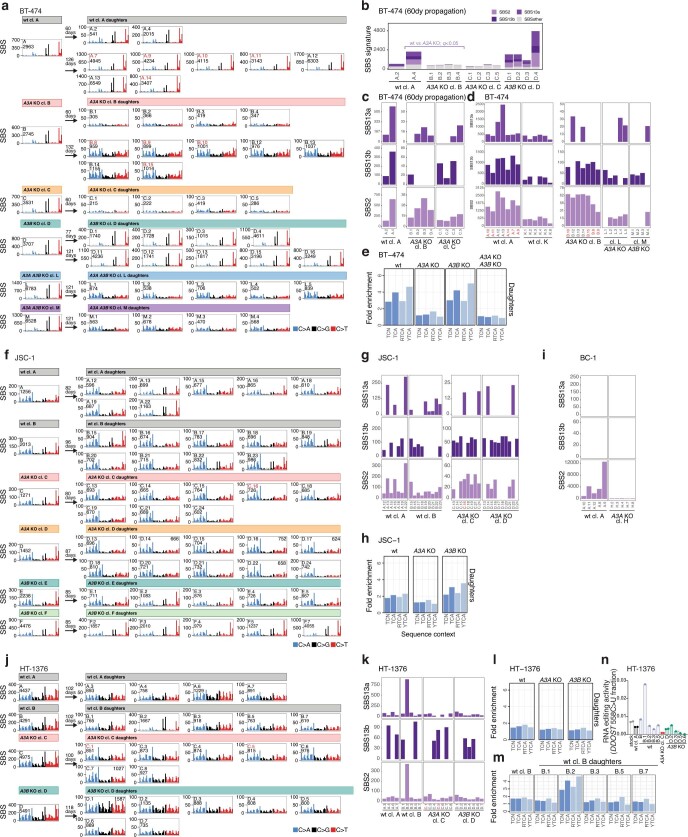
APOBEC3 deaminases drive acquisition of SBS2 and SBS13 in human cancer cells. **a**,**f**,**j)** Mutational profiles of indicated clones plotted as numbers of genome-wide substitutions (y-axis) at cytosine bases classified into 48 possible trinucleotide sequence contexts (x-axis; detailed in Extended Data Fig. 4a). Arrows indicate the number of days spanning the cloning events of parents (left of arrow) and daughters (right), during which mutation acquisition was tracked. **b)** Numbers of SBS attributed to colour-coded mutational signatures discovered in indicated daughter clones. *Q*-values were calculated using one-tailed Mann-Whitney *U-*tests and FDR corrected using the Benjamini-Hochberg procedure.**c**,**d**,**g**,**i**,**k)** Focused plots showing indicated SBS2, SBS13a/b burdens in indicated daughter clones. **e**,**h**,**l**,**m)** Enrichment of cytosine mutations at APOBEC3B-preferred RTCA and APOBEC3A-preferred YTCA sequence contexts (R = purine base, Y = pyrimidine base, N = any base) in daughter clones from indicated cell lines and genotypes. **m)** Quantification of *DDOST* 558C>U levels in the indicated HT-1376 cells. Bars represent the mean of 3 technical replicates and *n* = 2 experiments. Clones marked in red across panels were excluded from statistical tests (Methods).

**Extended Data Fig. 7:**
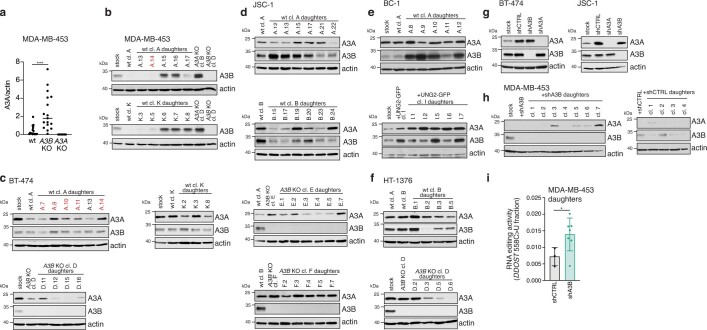
Characterization of APOBEC3A and APOBEC3B expression levels. **a)** Quantification of APOBEC3A protein levels relative to corresponding actin signals in the indicated daughter clones as shown in Fig. 3k (Mean, ***p = 0.0003, Student’s *t*-test, *n* = 2 experiments). **b-h)** Immunoblotting with anti-APOBEC3A (01D05), anti-APOBEC3B, and anti-actin antibodies in the indicated cell lines. **i)** Quantification of *DDOST* 558C>U levels in the indicated MDA-MB-453 cells. Bars represent the mean ± SD of *DDOST* 558C>U RNA editing activity across daughter clones. Data are derived from 3 shCTRL and 7 shA3B daughters across 3 technical replicates and *n* = 1 experiment. *P*-values were calculated using two-tailed Student’s t-test with Welch’s correction (*p < 0.05).

Ongoing generation of APOBEC3-associated SBS2 and SBS13a/b, SBS other, indels and chromosomal rearrangements were detected in wild-type clones from all of the cell lines (Fig. 3a–g and Extended Data Figs. 5 and 6a,b,f,j). The numbers of acquired SBS2 and SBS13a/b signatures, in contrast to other SBS mutations, varied across individual wild-type daughter clones derived from the same parent, consistent with previously reported episodic acquisition of these signatures in cancer cell lines^4^. For example, BC-1 daughter A.9 acquired 12,504 SBS2 mutations in 108 days, whereas daughter A.10, which was propagated in parallel and derived from the same parent clone, exhibited only 954 SBS2 mutations (Fig. 3f). The variations in SBS2 and SBS13a/b burdens could not be explained by multiclonality, perturbed cell growth or expression level of candidate mutators (Extended Data Fig. 4b–d and Extended Data Fig. 7a–f).

An analysis of extended sequence contexts revealed an enrichment of cytosine mutations in APOBEC3A-preferred YTCA contexts in wild-type daughter clones from BRCA and BCL cell lines (Fig. 3i,j and Extended Data Fig. 6e,h). Most wild-type clones from the BLCA HT-1376 cell line acquired substantially lower SBS2 and SBS13a/b burdens compared with wild-type clones from other cell lines and exhibited a minor preference for APOBEC3B-preferred RTCA contexts (Fig. 3g and Extended Data Fig. 6l). This pattern resembled the RTCA mutation sequence contexts observed in a smaller proportion of cancers, which generally exhibit lower burdens of APOBEC3-associated mutations^23^.

Despite low expression and activity, *APOBEC3A* deletion significantly diminished acquisition of SBS2 and SBS13a/b mutations in BRCA and BCL cell lines (*q* values < 0.05 across all cell lines; Mann–Whitney *U*-tests; Fig. 3a–f and Extended Data Fig. 6a,b,f). The reduction in SBS2 and SBS13a/b was accompanied by a loss of the enrichment of mutations at YTCA sequences, demonstrating that previous observations of APOBEC3A sequence preferences in yeast^23^ can be extended to endogenous APOBEC3A activity in human cancer cells (Fig. 3i,j and Extended Data Fig. 6e,h). BLCA *APOBEC3A*-knockout clones from the HT-1376 cell line did not exhibit a significant decrease in SBS2 and SBS13a/b (Fig. 3g). Notably, a single wild-type daughter clone, HT-1376 B.2, exhibited a ninefold increase in SBS2 and SBS13a/b mutations and an enrichment of mutations in YTCA contexts, whereas other wild-type daughters possessed much smaller amounts of SBS2 and SBS13a/b mutations and enrichment of mutations in the RTCA sequence context (Fig. 3g and Extended Data Fig. 6k,m). The increase in mutations accompanied by a shift towards APOBEC3A-preferred motifs in this clone is consistent with APOBEC3A-associated episodic bursts of mutagenesis^4^. Overall, the inability to detect differences after *APOBEC3* deletion in BLCA cell lines may derive from a lack of power to capture or quantify rare APOBEC3 mutagenesis in wild-type clones (Methods). An increase in APOBEC3A-associated RNA-editing activity was not detected in HT-1376 B.2 cells relative to other wild-type daughters (Extended Data Fig. 6n). Thus, RNA-editing assays may not capture intermittent APOBEC3 activities, while shifting sequence context preferences across lineages complicate simple classification on the basis of enrichment of cytosine mutations at these motifs.

Surprisingly, despite higher expression and deaminase activity of APOBEC3B compared with APOBEC3A in all of the cell lines, deletion of *APOBEC3B* did not significantly reduce SBS2 and SBS13a/b burdens in cell lines with strong APOBEC3 mutagenesis (Fig. 3a–f and Extended Data Fig. 6a,b,f,j). Taken together, these results demonstrate that APOBEC3A is a main driver of SBS2 and SBS13 in BRCA and BCL cell lines, challenging inferences derived from high APOBEC3B expression and catalytic activity in extracts^14,15^.

## APOBEC3B in SBS2 and SBS13 generation

Although deletion of *APOBEC3B* did not significantly reduce acquisition of SBS2 and SBS13a/b, strong underlying activity of APOBEC3A in BRCA and BCL cell lines may obscure small differences in mutation burdens between wild-type and *APOBEC3B-*knockout daughters. Indeed, although strongly diminished, ongoing acquisition of SBS2 and SBS13a/b was detected in *APOBEC3A-*knockout daughter clones, indicating that additional APOBEC3 member(s) may be operative (Fig. 3a–f,h and Extended Data Fig. 6a–d,f–i). Furthermore, such mutations were accompanied by a shift in the enrichment from APOBEC3A-preferred YTCA observed in wild-type clones to APOBEC3B-associated RTCA sequence contexts in *APOBEC3A*-knockout clones from BRCA and BCL cell lines (Fig. 3i,j and Extended Data Fig. 6e,h). To investigate whether APOBEC3B generates smaller burdens of SBS2 and SBS13a/b, we generated *APOBEC3A/APOBEC3B* double-knockout clones from BRCA cell lines (Extended Data Fig. 1). The knockout daughters from both cell lines acquired significantly fewer SBS2 and SBS13a/b burdens compared with the *APOBEC3A*-knockout counterparts (*q* values < 0.05; Fig. 3a,c,d,h and Extended Data Fig. 6a,d) confirming that APOBEC3B contributes small amounts of SBS2 and SBS13a/b mutations. Although further diminished, SBS2 and SBS13a/b burdens were not eliminated in all *APOBEC3A/APOBEC3B-*knockout daughters (Fig. 3a,c,d,h and Extended Data Fig. 6a,d). Given the small number of mutations detected in *APOBEC3A/APOBEC3B* knockouts, we cannot dismiss the possibility that SBS2 and SBS13a/b burdens are overestimated during mutational signature quantification (Methods). However, other features indicative of APOBEC3 mutagenesis were apparent in some clones (Methods), including APOBEC3-associated mutations in TCN contexts (Fig. 3a, for example, MDA-MB-453 clone L.10), further suggesting persistent APOBEC3 mutagenesis after *APOBEC3A/APOBEC3B* loss. Both BRCA cell lines carry APOBEC3H haplotype I (Methods), previously associated with increased mutational burdens in a small number of cancers with the *APOBEC3B* deletion polymorphism^31^. Thus, APOBEC3H or another APOBEC enzyme may contribute small amounts of APOBEC3 signatures in these cell lines.

Surprisingly, *APOBEC3B*-knockout daughters from the BRCA MDA-MB-453 cell line exhibited significantly more SBS2 and SBS13a/b mutations compared with their wild-type counterparts (*q* < 0.01; Fig. 3a,c). SBS2 and SBS13a/b reduction in MDA-MB-453 *APOBEC3A/APOBEC3B* double knockouts confirmed that this increase was caused by APOBEC3A-mediated mutagenesis (Fig. 3c). The increase in SBS2 and SBS13a/b burdens was not observed in BT-474 and HT-1376 *APOBEC3B-*knockout daughters, while the apparent increase in JSC-1 cells was driven by one out of two available *APOBEC3B*-knockout lineages (Fig. 3d,e,g). Increased SBS2 and SBS13a/b mutation burdens were associated with stabilized APOBEC3A protein levels across *APOBEC3B-*knockout daughters from MDA-MB-453 compared with wild-type counterparts (Fig. 3k and Extended Data Fig. 7a,b). This effect was not observed in JSC-1, HT-1376 or BT-474 cells (Extended Data Fig. 7c,d,f).

To further assess whether heightened APOBEC3A mutagenesis in the absence of APOBEC3B may result from increased APOBEC3A protein levels, we used short hairpin RNA (shRNA) treatments to deplete APOBEC3B from stock cultures while avoiding clonal bottlenecking effects. The results confirmed that APOBEC3A protein levels were increased after *APOBEC3B* depletion in MDA-MB-453 and BC-1, but not BT-474 or JSC-1 cells (Fig. 3l and Extended Data Fig. 7g). APOBEC3A protein levels exhibited similar increases across daughter clones isolated from shAPOBEC3B-depleted parents (Extended Data Fig. 7h). APOBEC3B depletion also increased APOBEC3A-associated RNA-editing activity at *DDOST* transcripts in MDA-MB-453 stock cultures and in daughter clones isolated from shAPOBEC3B-depleted parents (*P* < 0.05; Student’s *t*-test; Fig. 3m and Extended Data Fig. 7i), further confirming that APOBEC3B depletion can increase APOBEC3A activity. APOBEC3A depletion did not affect APOBEC3B protein levels (Fig. 3l and Extended Data Fig. 7). Taken together, these results indicate that *APOBEC3B* loss can increase APOBEC3A protein levels, activity and mutagenesis in some cancer cells.

## APOBEC3s in kataegis and omikli

**Fig. 4:**
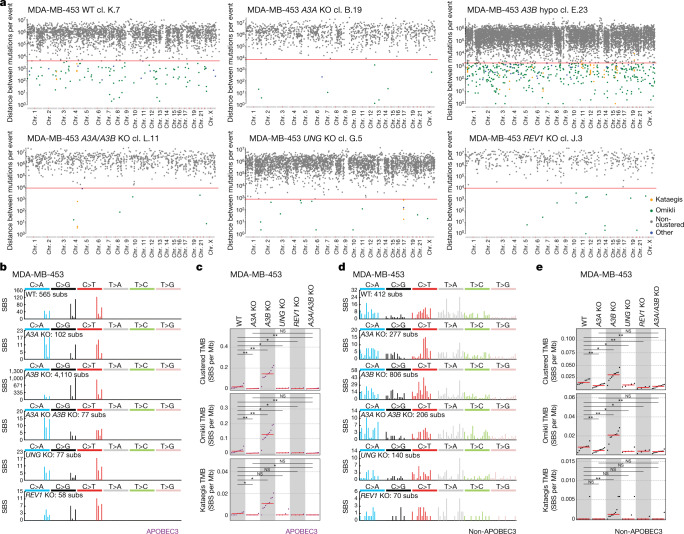
APOBEC3 deaminases drive the acquisition of clustered mutations in human cancer cells. **a**, Rainfall plots of the mutations acquired during in vitro propagation with each dot representing the distance between two SBSs. Dots are colour-coded on the basis of cluster type. log_10_-transformed intermutation distances are plotted on the *y* axes. The red lines represent sample-dependent intermutation distance cut-offs for detecting clustered mutations (Methods). **b**,**d**, Mutation spectra of clustered mutations in APOBEC3-associated (**b**) and non-APOBEC3-associated (**d**) contexts acquired in daughter clones from the indicated cell lines and genotypes. Mutational profiles plotted as the numbers of clustered genome-wide substitutions (subs) (*y* axis) at cytosine or thymine bases classified into 96 possible trinucleotide sequence contexts (*x* axis; Extended Data Fig. 4a). **c**,**e**, Clustered tumour mutational burdens (TMB), defined as numbers of total, kataegis and omikli APOBEC3-associated (**c**) (purple; cytosine mutations at TCN contexts) and non-APOBEC3-associated (**e**) (black; all other mutations) clustered SBSs per megabase in the indicated daughter clones. The red bars indicate the median tumour mutational burden. *q* values were calculated using two-tailed Mann–Whitney *U-*tests and were FDR-corrected using the Benjamini–Hochberg procedure; **q < 0.01, *q < 0.05; ns, not significant. Daughter clones with high proportions of shared mutations (Methods) were excluded from representation and statistical tests in **c** and **e**. Only mutations unique to individual daughter clones were considered in the representations in **b** and **d**.

**Extended Data Fig. 8:**
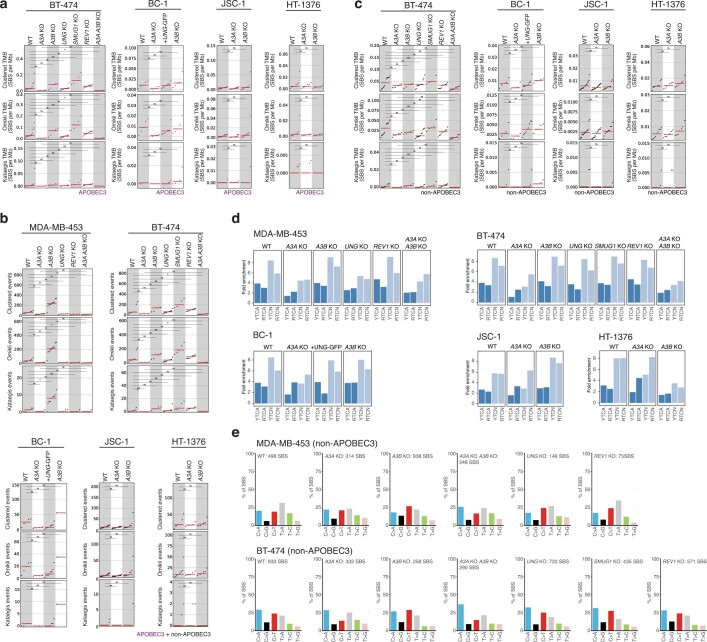
APOBEC3 deaminases drive acquisition of *kataegis* and *omikli* in human cancer cells. Clustered tumour mutational burdens (TMB), defined as numbers of total, *kataegis* and *omikli* **a)** APOBEC3-associated (purple; cytosine mutations at TCN contexts) and **c)** non-APOBEC3-associated (black; all other mutations) clustered SBS per megabase, in indicated daughter clones. Red bars indicate median TMB. **b)** Clustered TMB, defined as numbers of total, *kataegis* and *omikli* clustered genome-wide events, in indicated daughter clones. *q*-values (panels **a-c**) were calculated using two-tailed Mann-Whitney *U-*tests and FDR corrected using the Benjamini-Hochberg procedure (**q < 0.01; *q < 0.05; ns, not significant). **d)** Enrichment of clustered cytosine mutations at APOBEC3B-preferred RTCA and APOBEC3A-preferred YTCA sequence contexts (R = purine base, Y = pyrimidine base, N = any base) in daughters from indicated cell lines and genotypes. **e)** Mutational spectra of clustered mutations in non-APOBEC3-associated contexts acquired *de novo* in designated clones. Clones with high proportions of shared mutations (Methods) were excluded from representation and statistical tests in panels **a**-**c**. Only mutations unique to individual daughter clones were considered in representations in panel **e**.

The endogenous origins of APOBEC3-associated kataegis, that is, focal strand-coordinated hypermutation^1^, and omikli, that is, diffuse hypermutation^8^, have not been established in human cancer cells. Recent analyses showed that APOBEC3-associated signatures account for >80% of kataegis and >15% of omikli mutations in human cancers^11,22^. Kataegis burdens positively correlate with *APOBEC3B* expression levels^22^ and APOBEC3B can induce kataegis in an in vitro model of telomere crisis^32^. BRCA cell lines and, to a lesser degree, BCL and BLCA cell lines acquired de novo kataegis and omikli during in vitro propagation (Fig. 4, Extended Data Fig. 8a–c and Supplementary Table 6). The majority of these clusters primarily consisted of APOBEC3-associated cytosine mutations in TCN contexts, whereas others consisted of a more varied spectrum of mutations (Fig. 4b–e and Extended Data Fig. 8a,c). Mutation enrichment in YTCA/RTCA motifs was similar across clustered and genome-wide mutations in individual cell lines (Extended Data Fig. 8d).

*APOBEC3A* deletion significantly reduced burdens of clustered APOBEC3-associated mutations (*q* < 0.01; Mann–Whitney *U*-tests), including kataegis (*q* < 0.05) and omikli (*q* < 0.01) in MDA-MB-453 cells (Fig. 4b,c). Enrichment of mutations in YTCA contexts was diminished after *APOBEC3A* deletion (Extended Data Fig. 8d). Similar trends were observed in the BT-474 cell line (Extended Data Fig. 8a,d). Consistent with the increased burdens of SBS2 and SBS13a/b observed in *APOBEC3B*-deleted clones (Fig. 3a,c), there was an increased number of clustered APOBEC3-associated mutations (*q* < 0.01), including kataegis (*q* < 0.05) and omikli (*q* < 0.01), in MDA-MB-453 *APOBEC3B*-knockout clones (Fig. 4b,c). Small numbers of APOBEC3-like omikli mutations were detected in some *APOBEC3A/APOBEC3B* double-knockout clones from both MDA-MB-453 and BT-474 cells further suggesting that an additional APOBEC enzyme or mutagenic process may be operative (Fig. 4a–c and Extended Data Fig. 8a). Taken together, these data mirror observations of genome-wide mutations indicating that APOBEC3A accounts for the vast majority of kataegis and omikli mutation clusters in BRCA cells.

Unexpectedly, the loss of *APOBEC3A* caused a reduction in omikli mutations (*q* < 0.01) that occurred outside of APOBEC3-associated sequence contexts in MDA-MB-453 cells, whereas loss of *APOBEC3B* caused an increase in omikli (*q* < 0.01) and kataegis (*q* < 0.01) mutations falling outside these contexts (Fig. 4d,e). BT-474 cells exhibited similar trends that fell short of significance (Extended Data Fig. 8c). These mutations were broadly distributed across cytosine and thymine bases and did not display any detectable bias towards specific sequence contexts (Fig. 4d and Extended Data Fig. 8e). The precise origins of these mutations remain unknown, but they may derive from mutagenic TLS activity occurring at single-stranded DNA gaps^33^.

## DNA glycosylases in APOBEC3 mutagenesis

SBS2 is characterized by C>T mutations, whereas SBS13 consists of C>G and C>A mutations^10^. Processing of APOBEC3-generated uracil may dictate the resulting mutation type. On the basis of models of the processing of AID-mediated uracil during somatic hypermutation at immunoglobulin loci, replication across uracil is assumed to give rise to C>T mutations and, therefore, possibly SBS2^3,34,35^. Uracil excision by a glycosylase, such as UNG or SMUG1, followed by TLS may give rise to C>T, C>G and C>A mutations and therefore a combination of SBS2 and SBS13^3,34,35^. Indeed, genome-wide transversion mutations in yeast AID-overexpression models largely depend on UNG^36^. While BT-474, MDA-MB-453, JSC-1 and HT-1376 cells carry patterns of both SBS2 and SBS13a/b, BC-1 cells display only the SBS2 signature (Figs. 1b and 3c–g). This phenomenon was attributed to attenuated expression of the uracil glycosylase *UNG* due to *UNG* promoter methylation in BC-1^4^. Thus, uracil excision is predicted to be a critical mediator of APOBEC3 mutagenesis in human cancer cells.

**Fig. 5:**
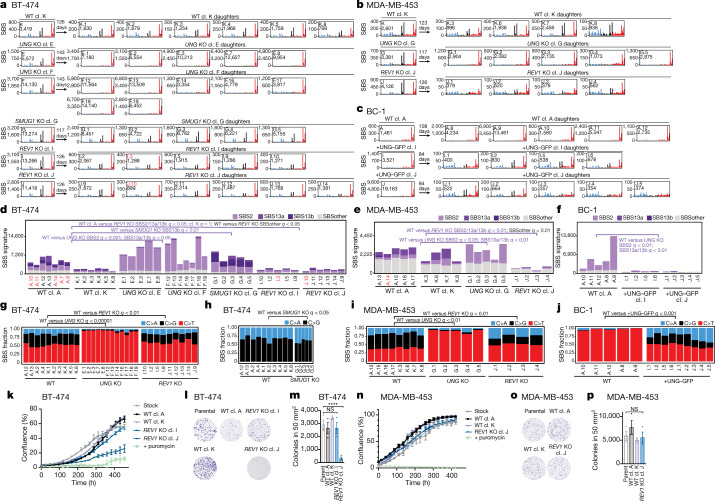
UNG and REV1 have critical roles in the generation of APOBEC3 mutations in cancer. **a**–**c**, Mutational profiles of the indicated BT-474 (**a**), MDA-MB-453 (**b**) and BC-1 (**c**) clones plotted as the numbers of genome-wide substitutions (*y* axis) at cytosine bases classified into 48 possible trinucleotide sequence contexts (*x* axis; Extended Data Fig. 4a). The arrows represent the number of days spanning the cloning events of parents (left from the arrow) and daughters (right) during which mutation acquisition was tracked. **d**–**f**, The numbers of SBSs attributed to colour-coded mutational signatures discovered in daughter clones from the indicated BT-474 (**d**), MDA-MB-453 (**e**) and BC-1 (**f**) cell lines and genotypes. SBSs from wild-type daughters were duplicated from Fig. 3c,d,f to facilitate the comparison. **g**–**j**, The proportions of the indicated mutation types in TCN contexts in the indicated BT-474 (**g**,**h**), MDA-MB-453 (**i**) and BC-1 (**j**) clones. *q* values indicate the differences between the indicated experiments in the proportions of C>A and C>G mutations (**g**,**i**,**j**) or C>G mutations (**h**). Only clones that were otherwise considered in statistical analyses are shown (Methods). *q* values (**d**–**j**) were calculated using one-tailed Mann-Whitney *U-*tests and FDR-corrected using the Benjamini–Hochberg procedure. **k**,**n**, Confluency measurements of the indicated cell lines. Data are mean ± s.d. of three technical replicates. Each experiment is representative of *n* = 3 biological replicates. **l**,**o**, Clonogenic survival of the indicated BT-474 (**l**) and MDA-MB-453 (**o**) cell lines. **m**,**p**, Quantification of clonogenic survival as in **l** and **o**. Data are mean ± s.d. Statistical analysis was performed using one-way ANOVA with Tukey multiple-comparison test; ****P < 0.0001. *n* = 3 experiments.

To directly assess the effect of UNG and SMUG1 on the generation of SBS2 and SBS13a/b in cancer cells, we expressed UNG–GFP in BC-1 cells, and CRISPR–Cas9 deleted *SMUG1* from BT-474 cells and *UNG* from BT-474 and MDA-MB-453 cells (Extended Data Figs. 1k and 3a–c,g–j). Deletion of *UNG* reduced the relative proportions of C>A and C>G mutations in TCN contexts in daughter clones from BRCA cell lines (*q* values < 0.01), while GFP-UNG expression in BC-1 cells increased the proportion of those mutation types in BC-1 daughter clones (*q* < 0.001) (Fig. 5a–c,g,i,j). Consistent with these data, SBS13a/b mutations decreased, but were not eliminated, in *UNG*-knockout clones from both cell lines (*q* values < 0.05) and appeared in BC-1 cells reconstituted with UNG–GFP (*q* < 0.01) (Fig. 5d–f). Thus, consistent with observations that UNG can excise AID-mediated uracil^35–37^, these results indicate that UNG connects genome-wide APOBEC3 deaminase activity to transversion mutations.

*UNG* deletion led to decreases in APOBEC3-associated mutations and other overall clustered mutations in MDA-MB-453 cells (*q* values < 0.05; Fig. 4c,e). The precise mechanism linking UNG activity to clustered mutations will require further investigation, but may involve APOBEC3 and TLS activities at single-stranded DNA exposed during homologous recombination or mismatch-repair-associated DNA end resection at UNG-initiated DNA breaks^36^.

*SMUG1* deletion resulted in a higher proportion of C>A mutations relative to C>G mutations in TCN contexts (*q* < 0.05) and an increase in SBS13b (*q* < 0.01), which is characterized by a higher proportion of C>A mutations relative to C>G in SBS13a (Fig. 5d,h and Supplementary Table 4). Considered with the persistent C>G/A mutations observed in *UNG-*knockout daughter clones (Fig. 5d–f), these results suggest that SMUG1 may excise APOBEC3-mediated uracil bases, consistent with previous observations indicating that SMUG1 can occasionally substitute for UNG in the repair of U:G lesions^38^.

## *REV1* in cells with APOBEC3 mutagenesis

To assess the contribution of TLS to the generation of SBS2 and SBS13a/b, we deleted *REV1*—a TLS polymerase with deoxycytidyl transferase activity opposite abasic sites^39^—from BRCA cell lines (Extended Data Fig. 3d–f,j). *REV1* deletion led to a decrease in SBS2 and SBS13a/b mutations in *REV1-*knockout daughters of MDA-MB-453 cells (*q* < 0.01) compared with wild-type clones, and in knockout daughters from BT-474 cells compared with clones from one (A; *q* < 0.05), but not the other, wild-type lineage (K; *q* = 1), which acquired substantially lower numbers of mutations (Fig. 5a,b,d,e). The relative proportion of C>G mutations in TCN contexts was reduced in *REV1*-knockout daughter clones from both cell lines compared with their wild-type counterparts (*q* values < 0.01; Fig. 5g,i). Deletion of *REV1* in MDA-MB-453 cells also resulted in a significant decrease in clustered mutations occurring within APOBEC3-associated sequence contexts (*q* < 0.05; Fig. 4b,c). Consistent with the proposed roles of REV1 during AID-mediated mutagenesis^33,36,40–42^, reductions in C>G proportions in *REV1-*knockout daughters are likely to reflect a loss of REV1 deoxycytidyl transferase activity, whereas diminished burdens of SBS2 and SBS13a/b presumably derive from the loss of the non-catalytic role of REV1 in acting as a scaffold for the coordination of other Y-family polymerases. These results directly link REV1 to the generation of APOBEC3-mediated mutational signatures in human cancer cell genomes.

**Extended Data Fig. 9:**
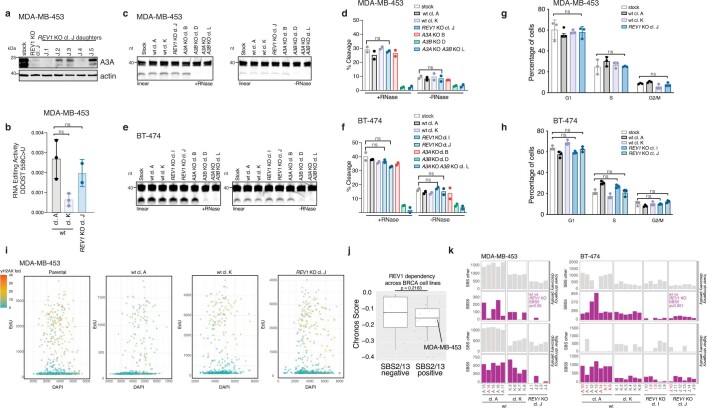
REV1 does not exhibit synthetic lethal interaction with APOBEC3. **a)** Immunoblotting with anti-APOBEC3A (04A04) and anti-actin antibodies in the indicated cell lines (*n* = 3 experiments). **b)** Quantification of *DDOST* 558C>U levels in the indicated MDA-MB-453 cells (Mean ± SD, ns, not significant, one-way ANOVA with Tukey’s multiple-comparisons test, *n* = 3 experiments). **c**,**e)** Cytosine deaminase activity in the indicated cell lines measured against linear probes ± RNase treatment to degrade RNA in extracts. **d**,**f)** Quantification of APOBEC3 deaminase activity as a percentage of processed DNA as in **c**,**e**) (Mean, ns, not significant, one-way ANOVA with Tukey’s multiple-comparisons test, *n* = 2 experiments). **g**,**h)** Plots showing cell cycle distribution of the indicated cell lines (mean ± SD, *n* = 3 experiments). **i)** γH2AX, EdU, and DAPI levels were quantified and plotted across the indicated axes. Dots represent individual cells that were coloured according to the intensity of γH2AX staining. **j)** DepMap CRISPR dependency data of 27 BRCA cell lines, classified as ‘SBS2/13 negative’ and ‘SBS2/13’ positive (Methods), on *REV1*. The dots represent cell lines plotted alongside the y-axis denoting the Chronos Dependency Score (Methods). The box represents the 25th-75th percentile of the data, centre line represents the median, the upper and lower whiskers indicate the maximum and minimum data points without considering boxplot outliers (larger dots, respectively, any values 1.5 times the interquartile range over the 75th or under the 25th percentile). *P*-value was calculated using a one-tailed Mann-Whitney *U* test. **k)** Focused plots showing indicated SBS5 and ‘SBS other’ burdens in the indicated cell lines in analyses where signatures were identified with lower or higher stringency discovery penalties (Methods). *q*-values were calculated using one-tailed Mann-Whitney *U*-tests and FDR corrected using the Benjamini-Hochberg procedure. Clones marked in red were excluded from statistical tests (Methods).

Beyond APOBEC3-associated mutations, burdens of other SBS and clustered mutations occurring outside of APOBEC3-associated sequence contexts were reduced, respectively, in *REV1*-knockout daughters from both BRCA cell lines (*q* values < 0.05; Fig. 5d,e) and MDA-MB-453 cells (*q* < 0.01; Fig. 4d,e). These observations are consistent with previous reports indicating that REV1 mediates a wide variety of SBS types^36,42,43^. Most signatures grouped into the ‘SBS other’ class were characterized by flat profiles and low mutational burdens, which challenge signature attribution (Methods). However, SBS5 was the only signature discovered consistently across wild-type clones from both cell lines (Supplementary Table 4). Given the relatively uniform distribution of 96 SBS classes in SBS5, we cannot exclude the possibility that the activities of SBS5 in individual clones are overestimated (Methods). However, the discovery of SBS5 across all wild-type clones is consistent with previous reports on SBS5 representing a signature of an unknown process operative continuously throughout life across all tissues^29,30^. SBS5 burdens were significantly depleted in *REV1*-knockout cells of the MDA-MB-453 and BT-474 cell lines (*q* values < 0.05; Extended Data Fig. 9k).

*REV1*-knockout cells did not consistently exhibit decreased proliferation, clonal survival, APOBEC3A protein levels or APOBEC3 catalytic activities (Fig. 5k–p, Extended Data Fig. 4c,d and Extended Data Fig. 9a–f). Furthermore, *REV1-*knockout cells did not exhibit altered cell cycle dynamics or increased DNA damage when compared to MDA-MB-453 and BT-474 stock cultures or wild-type subclones (Extended Data Fig. 9g–i). Finally, an analysis of available genome-wide drop-out CRISPR screens across cell lines with and without APOBEC3 signature mutations failed to show an increased dependence on REV1 in cancer cell lines containing SBS2/13 mutations (Methods and Extended Data Fig. 9j). Thus, diminished mutation burdens in the *REV1*-knockout cells could not be attributed to perturbed growth or clonal survival. Instead, these results indicate that REV1 has a critical role in the generation of both SBS2 and SBS13 and may contribute to the mutational process underlying SBS5.

## Discussion

Research in model systems and multiple associations has implicated APOBEC3 deaminases in cancer mutagenesis^3,13,44^. Here, by deleting candidate APOBEC3 mutators from human cancer cell lines that generate the relevant mutations naturally over time^4^, we provide causal evidence for the hypothesis put forward two decades ago that APOBEC3 enzymes can act as endogenous sources of mutation in cancer^6^. The results demonstrate that APOBEC3A is the major driver of clustered and non-clustered APOBEC3 mutational signatures in cancer cell lines in which results from surrogate assays of APOBEC3 activities recapitulated current debates in the field. Consistent with observations in yeast, endogenous APOBEC3A exhibits a preference for YTCA motifs, which account for a major proportion of APOBEC3 mutational signatures in cancer^23,25^. Future work will be necessary to dissect the mechanisms of APOBEC3 mutagenesis in cancers exhibiting an enrichment of genome-wide or clustered cytosine mutations in APOBEC3B-favoured RTCA motifs. Direct identification of APOBEC3A as a major generator of prevalent mutational signatures in cancer is a critical step forward for future studies seeking to define the underlying causes of APOBEC3 mutagenesis and to take advantage of APOBEC3 mutagenesis for therapeutic benefit. Our data demonstrate that APOBEC3B contributes a small number of mutations, therefore challenging previous predictions based on high *APOBEC3B* expression levels and deaminase activity, including in cell lines analysed here (that is, BT-474, MDA-MB-453) that APOBEC3B is the dominant mutator^14,15^. Our results demonstrate that APOBEC3A expression, activity and mutagenesis can be increased by the loss of APOBEC3B in some cancer cell lines. This result is reminiscent of the higher APOBEC3-associated mutation burdens observed in breast cancers that develop in carriers of a common germline deletion polymorphism that effectively deletes *APOBEC3B* and stabilizes the expression of the resulting *APOBEC3A*–*APOBEC3B* hybrid transcript^21,45^. However, the CRISPR edits used in our experiments do not resemble the features of this deletion polymorphism and are not predicted to generate a fusion transcript. Similar increases in *APOBEC3A* mRNA expression have previously been observed in BRCA cells after APOBEC3B depletion^24^. Thus, APOBEC3B may regulate APOBEC3A mutagenesis across a broader range of cancers, possibly through regulating the expression of APOBEC3A. Understanding the extent and mechanisms of this observation requires further investigation. Furthermore, our results imply that another APOBEC enzyme may contribute the relevant signatures in cancer.

Finally, our data directly link uracil excision by UNG and REV1-dependent TLS to the acquisition of APOBEC3-induced signatures in human cancer cells. Mutations associated with the activities of other TLS polymerases have been discovered in human genomes^9,46–49^. Consistent with the roles of REV1 in TLS^43^, endogenous REV1 activity contributed to acquisition of a broader spectrum of mutation types. Despite being one of the most prevalent signatures in cancer and normal tissues^29,30^, the mutational processes underlying the generation of SBS5 are largely unknown. Increased burdens of SBS5 in urothelial cancer have been associated with mutations in the *ERCC2* gene encoding a DNA helicase that has a central role in the nucleotide-excision repair pathway^50^. However, urothelial cancer is unique in that it is the only known tumour type in which the core nucleotide excision repair (NER) gene *ERCC2* is significantly mutated, whereas SBS5 activity has been identified in all tumour and normal tissues characterized to date^29,30^. Our results indicate that SBS5 may in part also represent a footprint of lower-fidelity REV1-dependent translesion synthesis.

## Methods

### Data reporting

No statistical methods were used to predetermine sample size. The investigators were not blinded to allocation during experiments and outcome assessment.

### Cell culture

MDA-MB-453, BT-474, JSC-1 and BC-1 cell lines were acquired from the cryopreserved aliquots of cell lines sourced previously from collaborators or public repositories and extensively characterized as part of the Genomics of Drug Sensitivity in Cancer (GDSC)^51,52^ and COSMIC Cell Line projects^4,53^. Bulk cell lines were genotyped by single-nucleotide polymorphism (SNP) and short tandem repeat profiling, as part of the COSMIC Cell Line Project (https://cancer.sanger.ac.uk/cell_lines) and individual clones obtained here were genotyped (Fluidigm) to confirm their accurate identities. MCF10A cells were from M. Jasin’s laboratory (MSKCC). HT-1376 cells were from B. Faltas’s laboratory (Weill Cornell). HEK293FT cells were from T. de Lange’s laboratory (Rockefeller).

All cell lines were mycoplasma negative (Mycoalert Detection Kit; Lonza). MDA-MB-453 cells were grown in DMEM:F12 medium supplemented with 10% fetal bovine serum (FBS) and 1% penicillin–streptomycin. BC-1, BT-474 and JSC-1 cells were grown in RPMI medium supplemented with 10% FBS, 1% penicillin–streptomycin, 1% sodium pyruvate and 1% glucose. HT-1376 cells and HEK293FT cells were grown in DMEM HG medium supplemented with 10% FBS and 1% penicillin–streptomycin. MCF10A cells were cultured in 1:1 mixture of F12:DMEM medium supplemented with 5% horse serum (Thermo Fisher Scientific), 20 ng ml^−1^ human EGF (Sigma-Aldrich), 0.5 mg ml^−1^ hydrocortisone (Sigma-Aldrich), 100 ng ml^−1^ cholera toxin (Sigma-Aldrich) and 10 μg ml^−1^ recombinant human insulin (Sigma-Aldrich). Unless otherwise noted, all media and supplements were supplied by the MSKCC Media Preparation core facility.

### Generation of knockout cell lines

Cells (10^6^) were electroporated using the Lonza 4D-Nucleofector X Unit (MDA-MB-453) or Lonza Nucleofector 2b Device (BT-474, BC-1, JSC-1, HT-1376) using programs DK-100 (MDA-MB-453), X-001 (BT-474, HT-1376) or T-001 (BC-1, JSC-1) in buffer SF + 18% supplement (MDA-MB-453) or 80% solution 1 (125 mM Na_2_HPO_4_•7H_2_O, 12.5 mM KCl, acetic acid to pH 7.75) and 20% solution 2 (55 mM MgCl_2_) (BT-474, BC-1, JSC-1, HT-1376) and 9 µg (UNG, SMUG1, REV1) or 10 µg (APOBEC3A, APOBEC3B) of pU6-sgRNA_CBh-Cas9-T2A-mCherry plasmid DNA (Supplementary Table 5). mCherry-positive cells were single-cell sorted or bulk sorted and subcloned by limited dilution into 96-well plates by FACS using the FACSAria system (BD Biosciences).

### Knockout screening and validation by PCR

#### CRISPR knockout clone screening

Genomic DNA was isolated using the Genomic DNA Isolation Kit (Zymo Research; ZD3025). Purified genomic DNA for CRISPR–Cas9 knockout screens was amplified using Touchdown PCR. Each PCR reaction comprised 7.4 μl double-distilled H_2_O, 1.25 μl 10× PCR buffer (166 mM NH_4_SO_4_, 670 mM Tris base pH 8.8, 67 mM MgCl_2_, 100 mM β-mercaptoethanol), 1.5 μl 10 mM dNTPs, 0.75 μl DMSO, 0.25 μl forward and reverse primers (10 μM each), 0.1 μl Platinum Taq DNA Polymerase (Invitrogen; 10966083) and 1 μl genomic DNA. A list of primer sequences is provided in Supplementary Table 5.

#### PCR for Sanger sequencing

PCR reactions for Sanger Sequencing were performed using the Invitrogen Platinum Taq DNA Polymerase (Invitrogen, 10966083) protocol. Genomic DNA (25 ng) was used for each reaction. A list of the primer sequences is provided in Supplementary Table 5. DNA from PCR reactions was purified from agarose gels using the Invitrogen PureLink Quick Gel Extraction Kit (Invitrogen, K210012). Gel-purified DNA was cloned using the TOPO TA Cloning Kit for Sequencing (Invitrogen; 450030) and colonies were selected for sequencing (Genewiz).

### Lentiviral transduction

Lentiviral plasmids for APOBEC3A, APOBEC3B and control knockdown were provided by S. Roberts’ laboratory^24^. For UNG–GFP lentiviral transduction, UNG2 open reading frames were amplified from a BT-474 cDNA library using the Phusion High-Fidelity polymerase (Thermo Fisher Scientific) and Gibson (NEB) assembled into pLenti-CMV-GFP BlastR (Addgene). The constructs were transfected into HEK293FT cells together with psPAX2 and pMD2.G (Addgene) using calcium phosphate precipitation. Supernatants containing lentivirus were filtered and supplemented with 4 μg ml^−1^ polybrene. Successfully transduced BC-1 cells were selected by FACS and clones isolated by limiting dilution. For shRNA knockdown, after transduction, cells were selected with hygromycin B.

### RNA isolation and quantitative PCR

RNA was isolated using the Quick-RNA Miniprep Kit (Zymo Research; R1054). RNA was quantified and converted to cDNA using the SuperScript IV First-Strand Synthesis System (Invitrogen; 18091050). cDNA synthesis reactions were performed using 2 μl of 50 ng μl^−1^ random hexamers, 2 μl of 10 mM dNTPs, 4 μg RNA and DEPC-treated water to a volume of 26 μl. The mixture was heated at 65 °C for 5 min, then cooled on ice for 5 min. Primers, probes and cycling conditions were adopted from published methods^54^. A list of the primer sequences is provided in Supplementary Table 5.

### Immunoblotting

Cells were lysed in RIPA buffer (150 mM NaCl, 50 mM Tris-HCl pH 8.0, 1% NP-40, 0.5% sodium deoxycholate, 0.1% SDS, Pierce Protease Inhibitor Tablet, EDTA free) or sample buffer (62.5 mM Tris-HCl pH 6.8, 0.5 M β-mercaptoethanol, 2% SDS, 10% glycerol, 0.01% bromophenol blue). Quantification of RIPA extracts was performed using the Thermo Fisher Scientific Pierce BCA Protein Assay kit. Protein transfer was performed by wet transfer using 1× Towbin buffer (25 mM Tris, 192 mM glycine, 0.01% SDS, 20% methanol) and nitrocellulose membrane. Blocking was performed in 5% milk in 1× TBST (19 mM Tris, 137 mM NaCl, 2.7 mM KCl and 0.1% Tween-20) for 1 h at room temperature. The following antibodies were diluted in 1% milk in 1× TBST: anti-APOBEC3A/B/G (04A04) and anti-APOBEC3A (01D05) (see below; western blot, 1:1,000), anti-APOBEC3B (Abcam; ab184990; western blot, 1:500), anti-REV1 (Santa Cruz; sc-393022, western blot, 1:1000), anti-SMUG1 (Abcam; ab192240; western blot, 1:1,000 and Santa Cruz; sc-514343; western blot, 1:1,000), anti-UNG (abcam; ab109214; western blot, 1:1,000), anti-GFP (Santa Cruz; sc-9996; western blot, 1:1,000), anti-β-actin (Abcam; ab8224; western blot, 1:3,000), anti-β-actin (Abcam, ab8227; western blot, 1:3,000); anti-mouse IgG HRP (Thermo Fisher Scientific; 31432; 1:10,000), anti-rabbit IgG HRP (SouthernBiotech; 6441-05; 1:10,000).

### APOBEC3 monoclonal antibody generation

Residues 1–29 (N1-term) or 13–43 (N2-term) from APOBEC3A and residues 354–382 (C-term) from APOBEC3B and were used to create three peptide immunogens (EZBiolab). Five mice were given three injections using keyhole limpet haemocyanin (KLH)-conjugated peptides over the course of 12 weeks (MSKCC Antibody and Bioresource Core). Test bleeds from the mice were screened for anti-APOBEC3A titres by enzyme-linked immunosorbent assay (ELISA) against APOBEC3A peptides conjugated to BSA. Mice showing positive anti-APOBEC3A immune responses were selected for a final immunization boost before their spleens were collected for B cell isolation and hybridoma production. Hybridoma fusions of myeloma (SP2/IL6) cells and viable splenocytes from the selected mice were performed by the MSKCC Antibody and Bioresource Core. Cell supernatants were screened by APOBEC3A ELISA. The strongest positive hybridoma pools were subcloned by limiting dilution to generate monoclonal hybridoma cell lines. The hybridomas 04A04 (anti-APOBEC3A/B/G) and 01D05 (anti-APOBEC3A) were expanded then grown in 1% FBS medium. This medium was clarified by centrifugation and then passed over a protein G column (04A04) or protein A column (01D05) to bind to monoclonal antibodies. The resulting monoclonal antibodies were eluted in PBS (04A04) or in 100 mM sodium citrate pH 6.0, 150 mM NaCl buffer and subsequently dialysed into PBS (01D05).

### Cell cycle and apoptosis assays

Annexin V staining was performed using the annexin V Apoptosis detection kit (BD Biosciences) according to the manufacturer’s instructions. For propidium iodide plus BrdU double staining, BrdU was added to the culture medium to a final concentration of 10 μM for 1 h. Cells were fixed with 70% ethanol and treated with 2 M hydrochloric acid for 20 min. BrdU staining was performed with 20 μl of anti-BrdU antibodies (25 μg ml^−1^, B44, Becton Dickinson) for 15 min at room temperature followed by a 15 min incubation with 50 μl Alexa Fluor 488 goat anti mouse at 40 μg ml^−1^ (Invitrogen). After a final wash, cells were taken up in 100 μg ml^−1^ PI with 20 μg ml^−1^ RNase A. Flow data were collected on the Fortesa or LSR-II analyzer and analysed using FlowJo v.10.

### Automatic counting of γH2AX foci

EdU staining was performed by using Click-iT EdU Alexa Fluor 488 Imaging Kits (Invitrogen, C10337) according to the manufacturer’s instructions. For EdU incubation, EdU was added to the culture medium to a final concentration of 10 μM for 2 h. Cells were fixed with 2% paraformaldehyde for 15 min at room temperature followed by 0.5% Triton X-100 permeabilization for 5 min. Click-iT reaction was performed according to the manufacturer’s instructions. γH2AX was stained with anti-γH2AX antibodies (EMD Millipore, 05-636-1, 1:1,000) for 2 h at room temperature followed by anti-mouse secondary antibody Alexa Fluor 647 (Invitrogen, A21235). Cells were stained with Hoechst (1 μg μl^−1^) and mounted with Prolong Gold Antifade Reagent (Invitrogen, P36934).

Images were acquired on the DeltaVision Elite system equipped with a DV Elite CMOS camera, microtitre stage, and ultimate focus module (*z* stack through the cells at 0.2 mm increments). All of the images were processed by maximal projection of the *z* stack image series using the softWoRx software and analysed by Fiji. After separating channels using the ImageJ Macro Batch Split Channels tool, nuclear masks were generated by Fiji Macro CLAIRE, whereby nuclei are identified by radius in the Hoechst channel, binary processed (filling holes and watershed) and applied with auto local threshold (Phansalkar). Nuclear EdU and Hoechst intensity values were collected by measuring the mean intensity within nuclear masks (ROI measurement). To identify γH2AX foci, images were processed with background subtraction and Gaussian blur. γH2AX foci were displayed in ‘find maximum’ with output ‘point selection’ with manually adjusted parameters. The number of nuclear γH2AX foci was calculated by dividing the total γH2AX intensity at the displayed points (within the nuclear masks) with the intensity of a single γH2AX focus. All ImageJ macro and R codes were shared by M. Ferrari (M. Jasin Laboratory; MSKCC).

### Proliferation assays, doubling times and confluence experiments

Cells were seeded in triplicate in either 24-well or 48-well plates at a low dilution (5,000 to 20,000 depending on plate size and stock cell line basal growth). Growth over time was then measured by calculating daily cell confluency using an IncuCyte Live-Cell Analysis Imager (Essen/Sartorius). The IncuCyte takes images of each well and analyses them by applying a predetermined mask to each image that distinguishes between an empty surface and a surface covered by cells. Once the mask has been applied, the program calculates the surface area occupied by cells and the percentage confluency. Images were taken every 24 h and technical replicates were averaged to generate the percentage confluence, which was then plotted across time to generate growth curves. Alternatively, population doublings were measured by cell counting (Beckman Coulter). Cells were seeded from 1 million to 2 million cells per plate in triplicate and then allowed to grow for 72 h before being collected and counted (Beckman Coulter). The cells were then seeded once more at the same seeding value as the first time point and allowed to grow for another 72 h before being counted once more. This continued for three cycles. Cell counts were used to calculate population doublings between each time point.

### In vitro DNA deaminase activity assay

Deamination activity assays were performed as described previously^55^. In brief, 1 million (or 2 million MDA-MB-453) cells were pelleted and lysed in buffer (25 mM HEPES, 150 mM NaCl, 1 mM EDTA, 10% glycerol, 0.5% Triton X-100, 1× protease inhibitor), sheared through a 28 1/2-gauge syringe, then cleared by centrifugation at 13,000*g* for 10 min at 4 °C. Deaminase reactions (16.5 µl cell extracts with 2 µl UDG buffer (NEB), ±0.5 µl RNase A (20 mg ml^−1^), 1 µl of 1 µM probe (linear, 5′IRD800/ATTATTATTATTATTATTATTTCATTTATTTATTTATTTA; or hairpin, 5′IRD800/ATTATTATTATTGCAAGCTGTTCAGCTTGCTGAATTTATT), and 0.3 µl UDG (NEB)) were incubated at 37 °C for 2 h followed by addition of 2 µl 1 M NaOH and 15 min at 95 °C to cleave abasic sites. Reactions were then neutralized with 2 µl 1 M HCl, terminated by adding 20 µl urea sample buffer (90% formamide + EDTA) and separated on a prewarmed 15% acrylamide/urea gel in 1× TBE buffer at 55 °C for 70 min at 100 V to monitor DNA cleavage. Gels were imaged by Odyssey Infrared Imaging System (Li-COR) and quantified using ImageJ.

### RNA-editing assay

*DDOST* 558C>U RNA-editing assays were performed as described previously with assistance from the MSKCC Integrated Genomics Operation^28^. Total RNA was extracted using the RNeasy Mini kit (Qiagen) according to the manufacturer’s instructions. After extraction, the RNA was reverse-transcribed using the High Capacity cDNA Reverse Transcription Kit (Thermo Fisher Scientific). cDNA (20 ng) along with primers purchased from Bio-Rad (10031279 and 10031276) for the target *DDOST*^*558C>U*^ amplification were mixed in PCR reactions in a total volume of 25 μl. Then, 20 μl of the reactions were mixed with 70 μl of Droplet Generation Oil for Probes (Bio-Rad) and loaded into a DG8 cartridge (Bio-Rad). A QX200 Droplet Generator (Bio-Rad) was used to make the droplets, which were transferred to a 96-well plate and the following PCR reaction was then run: 5 min at 95 °C; 40 cycles of 94 °C for 30s and 53 °C for 1 min; and finally 98 °C for 10 min. The QX200 Droplet Reader (Bio-Rad) was then used to analyse the droplets for fluorescence measurement of the fluorescein amidite (FAM) and hexachloro-fluorescein (HEX) probes. The data were analysed using the QuantaSoft analysis software (Bio-Rad) and gating was performed on the basis of positive and negative DNA oligonucleotide controls.

### Comparison of APOBEC3-associated mutational signatures in cell lines with cancer data

Annotations of mutational signatures across 1,001 human cancer cell lines and 2,710 cancers from multiple cancer types were published previously^4^. Where possible, we matched cancer and cell line cancer classes as described in Supplementary Table 1. Eventually, 780 cell lines and 1,843 cancers from matching types were used in analyses presented in Fig. 1a. Individual classes and samples per class used are listed in Supplementary Table 1, and the signature annotation was published previously^4^.

### Whole-genome sequencing

Genomic DNA was extracted from a total of 251 individual clones using the DNeasy Blood and Tissue Kit (Qiagen) and quantified with the Biotium Accuclear Ultra high-sensitivity dsDNA Quantitative kit using Mosquito LV liquid platform, Bravo WS and the BMG FLUOstar Omega plate reader. Samples were diluted to 200 ng per 120 μl using the Tecan liquid handling platform, sheared to 450 bp using the Covaris LE220 instrument and purified using Agencourt AMPure XP SPRI beads on the Agilent Bravo WS. Library construction (ER, A-tailing and ligation) was performed using the NEB Ultra II custom kit on an Agilent Bravo WS automation system. PCR was set up using Agilent Bravo WS automation system, KapaHiFi Hot start mix and IDT 96 iPCR tag barcodes or unique dual indexes (UDI, Ilumina). PCR included 6 standard cycles: (1) 95 °C for 5 min; (2) 98 °C for 30 s; (3) 65 °C for 30 s; (4) 72 °C for 1 min; (5) cycle from step 2 five more times; (6) 72 °C for 10 min. Post-PCR plates were purified with Agencourt AMPure XP SPRI beads on the Beckman BioMek NX96 or Hamilton STAR liquid handling platform. Libraries were quantified using the Biotium Accuclear Ultra high sensitivity dsDNA Quantitative kit using Mosquito LV liquid handling platform, Bravo WS and the BMG FLUOstar Omega plate reader, pooled in equimolar amounts on a Beckman BioMek NX-8 liquid handling platform and normalized to 2.8 nM ready for cluster generation on a c-BOT system. Pooled samples were loaded onto the Illumina Hiseq X platform using 150 bp paired-end run lengths and sequenced to approximately 30× coverage, as described in Supplementary Table 1. Sequencing reads were aligned to the reference human genome (GRCh37) using Burrows–Wheeler Alignment (BWA)-MEM (https://github.com/cancerit/PCAP-core). Unmapped, non-uniquely mapped reads and duplicate reads were excluded from further analyses.

### Mutation identification

Somatic SBSs were discovered with CaVEMan (https://github.com/cancerit/cgpCaVEManWrapper)^56^, with the major and minor copy number options set to 5 and 2, respectively, to maximize discovery sensitivity. Rearrangements and indels were identified using BRASS (https://github.com/cancerit/BRASS) and cgpPindel^57^ (https://github.com/cancerit/cgpPindel), respectively. The sequences of the corresponding parent clones were used as reference genomes to discover mutations in individual daughter clones, whereas a sequence from an unrelated normal human genome^4^ (Supplementary Table 1) was used as a reference to discover mutations in parent clones. SBSs, indels and rearrangements were further filtered as described below. Comparisons performed and the numbers of mutations removed with individual filters are listed in Supplementary Table 1. SBSs, indel and rearrangement calls are available in Supplementary Tables 8–10.

SBSs discovered with CaVEMan were filtered over the six filters split into two steps: first, to remove the low-quality loci and, second, to ensure that the mutational catalogues from daughter clones retained exclusively mutations that were acquired during the relevant in vitro periods spanning the two cloning events and that the mutational catalogues from parent clones retained mutations unique to individual parent clones. SBSs shared between parent clones (see below) were used to derive proxies for the mutational catalogues of bulk cell lines (Fig. 1b).

First, only SBSs flagged by Caveman as ‘PASS’ when analysed against the panel of 98 unmatched normal samples (https://github.com/cancerit/cgpCaVEManWrapper)^56^ were considered, removing large proportions of mapping and sequencing artifacts, as well as the common germline variation^56^. Four post-hoc filters were applied to PASS variants to retain only mutations presenting at high-quality loci. SBSs were removed (1) if the median alignment score (ASMD) of mutation-reporting reads was less than or equal to 130; (2) if the mutation presented at a locus with the clipping index (CLPM) > 0; (3) if the mutation locus was covered by 15 or less reads in the reference samples used in comparisons; and (4) if mutations were not reported by at least one sequencing read of each direction.

Second, the remaining mutation loci were genotyped across all clones from the belonging cell lines. We used cgpVAF (https://github.com/cancerit/vafCorrect) to count the number of mutant and wild type reads across individual clones. Mutations were removed from each parent or daughter clone (5) if they presented in any reads of the corresponding reference samples or if (6) they presented in >50% of clones from other parental lineages from belonging cell lines. In mutational catalogues from parent clones, these steps served to remove the majority of the germline mutations and a smaller proportion of somatic mutations shared between parent clones, therefore retaining predominantly mutations unique to individual parent cell lineages acquired before the examined in vitro periods. In mutational catalogues from daughter clones, these steps served to remove small proportions of mutations (Supplementary Table 2) that were probably acquired before the examined periods in vitro that were not captured in the corresponding reference sequences. Mutations removed over these two steps were accumulated into approximate mutational catalogues of bulk cell lines (Fig. 1b). On average, only a small proportion of mutations was removed (~2%) with the final filter (6) from the daughter clones, pointing to a high-confidence ability to call de novo acquired mutations. Although these filters remove most of the germline and the pre-existing variation, a minor proportion of the removed mutations may have arisen independently across multiple parental lineages at the hairpin loci that are hotspots for APOBEC3-associated mutagenesis^26^.

This analysis revealed that, in rare instances, high proportions (>30%) of SBS mutations were shared between the related daughters and absent from their corresponding parents, indicating that such daughters were most likely established from a common subclone that arose during the cultivation of the parent clone. In total, 21 daughter clones (Supplementary Table 1; indicated in the relevant figures) were excluded from statistical comparisons relating to mutational burdens to ensure that considered daughter clones did not share high proportions of SBS.

Rearrangements and indels were identified only across daughter clones. Rearrangements that were not correctly reconstructed and were identified in the reference sequences by BRASS were removed. Indels were removed if they (1) presented at loci covered by 15 or less reads in the corresponding reference samples to ensure sequence coverage was sufficient to remove pre-existing mutations, (2) presented at only a single read in a considered sample to remove putative artifacts, (3) presented in any reads of a reference sample to ensure only mutations absent from the references were considered. Rearrangements and indels in daughter clones were further removed if they were detected in more than 50% of daughter clones from the related lineages to remove possibly pre-existing mutations.

### Validation of clonal sample origins

To ensure that samples were single-cell derived, we examined the proportions of the variant-reporting reads (equivalent to variant allele fraction (VAF)) at the mutation loci (Extended Data Fig. 4b). Consistent with the polyploid background of most of the cell lines under investigation^4^, VAF distributions often deviated from the average of ~50% expected for clonal heterozygous somatic mutations occurring in a diploid genome. The largely unimodal VAF distributions confirmed the clonal origins of the majority of the samples. On occasions in which bimodal VAF distributions were observed, at least one of the peaks followed the VAF distribution of all of the other related clones, indicating that the other peak originates from mutations acquired subclonally. Such instances were observed only in the BC-1 cell line.

### Sequence-context-based classification of single-base substitutions

SigProfilerMatrixGenerator^58^ (v.1.1; https://github.com/AlexandrovLab/SigProfilerMatrixGenerator) was used to categorize SBSs into three separate sequence-context based classifications. The algorithm allocates each SBS to (1) one of the 6 class categories (C>A, C>G, C>T, T>A, T>C and T>G) in which the mutated base is represented by the pyrimidine of the base pair; (2) to one of the 96 class categories (in which each of 6 class mutation types is further split into 16 subcategories on the basis of the 5′ and 3′ bases flanking the pyrimidine of the mutated base pair); (3) to one of the 288 class categories (in which each of 96 class mutation types is further split on the basis of whether it presents on the transcribed or untranscribed strand); and (4) to one of the 1,536 class categories (in which each of 6 class mutation types is further split into 256 subcategories on the basis of two 5′ and 3′ bases flanking the pyrimidine of the mutated base pair). The relevant outputs are shown in Supplementary Table 3.

### Enrichment of APOBEC3-associated mutations at trinucleotide and pentanucleotide motifs

Once SBSs were allocated to their sequence context classes as described, enrichment of C>T and C>G mutations was investigated across the APOBEC3-associated target trinucleotide motifs (TCN and TCA, where N is any base and the target base is underlined), and pentanucleotide motifs, which were previously associated with activities of APOBEC3A (YTCA, where Y is a pyrimidine base) and APOBEC3B (RTCA, where R is a purine base) in yeast overexpression systems^23^. C>A SBSs at TCN were not considered because those mutation types have been attributed to both APOBEC3-associated mutagenesis and other mutational processes arising during in vitro cell cultivation^4^.

Trinucleotide and pentanucleotide sequence motifs were quantified using sequence_utils (v.1.1.0, https://github.com/cancerit/sequence_utils/releases/tag/1.1.0;https://github.com/cancerit/sequence_utils/wiki#sequence-context-of-regions-processed-by-caveman) across regions of human autosomal chromosomes (GRCh37) that are considered by the CaVEMan algorithm in detecting SBSs. The middle base pair of each reference trinucleotide and pentanucleotide sequence was considered to be a putative mutation target and the surrounding sequence context was extracted by using the DNA strand belonging to the pyrimidine base of the target base pair. A total of 96 possible trinucleotide and 512 pentanucleotide contexts were quantified across both DNA strands (for example, the AGT trinucleotide is reported as ACT; the AAGCA pentanucleotide is reported as TGCTT). Enrichment of APOBEC3-associated mutations at the motifs of interest was calculated as described previously^4,23^. For example, to calculate enrichment (*E*) of cytosine mutations at RTCA sites the following was used:

$${E}_{{\rm{RT}}{\rm{C}}{\rm{A}}}=({{\rm{Mut}}}_{{\rm{RT}}{\rm{C}}{\rm{A}}}/{{\rm{Con}}}_{{\rm{RTCA}}})/({{\rm{Mut}}}_{{\rm{C}}}/{{\rm{Con}}}_{{\rm{C}}})$$

where Mut_RTCA_ is the total number of C>G and C>T mutations at RTCA contexts in autosomal chromosomes; Mut_C_ is the total number of C>G and C>T mutations in autosomal chromosomes; Con_RTCA_ and Con_C_ are the total numbers of available RTCA contexts and C bases, respectively. Enrichments of mutations in the other contexts, TCA, TCN and YTCA, were calculated analogously.

### Mutational signature analysis

Mutational signature analyses were performed over three steps using the SigProfilerExtractor tool (v.1.1.4; https://github.com/AlexandrovLab/SigProfilerExtractor)^59^. First, de novo signatures were extracted across 288-channel matrices (see the ‘Sequence-context-based classification of single-base substitutions’ section) of 1,317,120 genome-wide mutations from 5 bulk cell lines and 251 clones, using the non-negative matrix factorization (NNMF)-based function sig.sigProfilerExtractor, with factorizations between *k* = 1 and *k* = 20 signatures and over 500 iterations. This analysis revealed 10 signatures with an average stability of over 0.8, termed SBS288A-J (Supplementary Table 4). Second, the decomp.decompose function was used to match de novo identified mutational signatures to a set of reference set of COSMIC Signatures identified previously across more powered cancer datasets (v3.2; https://cancer.sanger.ac.uk/cosmic/signatures; Supplementary Table 4). This step enables distinguishing de novo signatures that have not been completely separated during the extraction and that can be explained by a combination of the known signatures from de novo signatures that have not been previously identified. Note that this step collapses 288-channel profiles of de novo identified signatures into 96-channel profiles to match the highly annotated 96-channel format of COSMIC signatures. SBS288A, SBS288B, SBS288C, SBS288F and SBS288H were successfully decomposed into a spectrum of known mutational signatures (cosine similarity > 0.97). Low-confidence decomposition (cosine similarity < 0.95) was achieved for SBS288D, SBS288E, SBS288G, SBS288I and SBS288J, indicating that these signatures probably represent new signatures that are absent from the COSMIC reference set. SBS288G was the only signature with low-confidence decomposition that was extracted over a low stability score (0.75) and was therefore not considered to be a new signature. SBS288E reflects patterns of SBS2 and SBS13, albeit with a higher relative proportion of C>A mutations in TCN contexts, and was therefore considered to be a new signature associated with APOBEC3-mediated deamination. SBS288I, SBS288J and SBS288D were considered new signatures of possibly unknown in vitro processes because they presented across multiple lineages of mostly individual cell lines and were not discovered previously across much larger sets of primary cancers from matching cancer types used to derive COMIC reference signatures. Third, the decomp.decompose function was used to quantify the activities of the final set of 96-channel mutational signatures, composed of the new and identified COSMIC signatures, across individual samples. Analyses were performed with default penalties for discovery of signatures in individual samples (results reported in Supplementary Table 4 (higher penalty)), as well as with lowered penalties (options ‘nnls_add_penalty’=0.005 and ‘nnls_remove_penalty’=0.001) to enable higher sensitivity of signature discovery (Supplementary Table 4 (lower penalty)). Manual inspection of mutational spectra of individual clones indicated that the higher discovery penalties increase the false-negative signature calls across the study. Thus, the signature estimations across individual samples displayed in the figures were performed using analyses with lowered discovery penalties.

Note that incorporation of the higher signature discovery penalties reduces the overall number of clones with APOBEC3-associated SBS2/13 in experiments in which their burdens are generally low, including in *APOBEC3A* knockouts of some cell lines, in double *APOBEC3A*/*APOBEC3B* knockouts and in wild-type and *APOBEC3A*- and *APOBEC3B*-knockout clones from the HT-1376 cell line. However, it does not change any of the findings pertaining to the SBS2/13 acquisition in those experiments (not shown). Despite this, we cannot exclude the possibility that SBS2/13 burdens may be overestimated in samples in which their overall burdens are low (<100 mutations). However, higher burdens of SBS2/13 (>100 mutations) have been detected among some, or multiple, clones from the indicated genotypes, consistent with persistent APOBEC3 mutagenesis. Moreover, reported NNMF-independent analyses, including analyses of clustered mutations in APOBEC3-associated sequence contexts, APOBEC3-associated spectrum of mutations in TCN contexts and enrichment of cytosine mutations at APOBEC3-associated cytosine mutations in TCN/TCA sequence contexts, further indicate that APOBEC3 mutagenesis is present or cannot be excluded in some, or multiple, clones from these genotypes.

Discovered signatures that are not APOBEC3-associated are signatures of flat profiles and/or low mutational burdens (Extended Data Fig. 4a and Supplementary Table 4) that challenge the accurate estimation of their activities in individual samples^60^ and/or were, after manual inspection, determined to probably be false-positive calls. This is further reflected in highly variable discovery of such signatures, with the exception of SBS5, in individual clones after different penalties used in signature discovery (Supplementary Table 4). Their activities were therefore summed for simplicity and represented together as ‘SBS_other’. SBS5 was accumulated into ‘SBS_other’, unless otherwise indicated. Given the general challenges associated with estimating the activities of signatures of flat profiles^60^, we cannot exclude the possibility that mutational burdens of SBS5 were overestimated in the study. However, analyses using both higher and lower penalties for signature discovery revealed a decrease in SBS5 after *REV1* knockout (Extended Data Fig. 9k).

### Identification of clustered mutations

To detect clustered SBSs, a sample-dependent intermutational distance (IMD) cut-off was derived, which is unlikely to occur by chance given the mutational pattern and mutational burden of each clone^11^. To derive a background model reflecting the distribution of mutations that one would expect to observe by chance, SigProfilerSimulator (v.1.1.2; https://github.com/AlexandrovLab/SigProfilerSimulator) was used to randomly simulate the mutations in each clone across the genome^61^. Specifically, the model was generated to maintain the ±1bp sequence context for each SBS, the strand coordination, including the transcribed or untranscribed strand within genic regions^58^, and the total number of mutations across each chromosome for a given sample. All SBSs were randomly simulated 100 times and used to calculate the sample-dependent IMD cut-off so that 90% of mutations below this threshold were clustered with respect to the simulated model (that is, not occurring by chance with a *q* value of <0.01). Furthermore, the heterogeneity in mutation rates across the genome were considered by correcting for mutation-rich regions present in 10-Mb-sized windows and by using a threshold for the difference in VAFs between subsequent SBSs in a clustered event (VAF difference < 0.10).

Identified clustered SBSs were categorized into specific classes: (1) omikli^8^ class, consisting of two or three mutations with all IMDs less than the sample-dependent IMD cut-off, at least a single IMD greater than 1 bp and consistent VAFs; (2) kataegis^1^ class, consisting of four or more mutations with all IMDs less than the sample-dependent IMD cut-off, at least a single IMD greater than 1 bp and with consistent VAFs; (3) doublet class, consisting of two adjacent mutations with consistent VAFs; (4) multibase class, consisting of three or more adjacent mutations with consistent VAFs. Doublet and multi-base classes, alongside all of the other clustered SBSs with inconsistent VAFs, were classified as ‘other’.

Classes were presented as both clustered SBSs (Fig. 4 and Extended Data Fig. 8a,c–e), which reflect single mutations, and clustered events (Extended Data Fig. 8b), which encompass the local grouping of clustered SBS (that is, a kataegis event encompasses four or more adjacent clustered SBS). For example, a single sample might have 5 kataegis events, with 6 SBSs per event, which would encompass a total of 30 SBSs. Clustered SBS tumour mutational burden was calculated using the total number of SBSs across a given clustered class, whereas the clustered event tumour mutational burden was calculated using the total number of events across a given clustered class. The combined clustered mutation tumour mutational burden was calculated by summing the total number of clustered SBSs or events across all subclasses. Clustered SBSs were further classified into 96-class categories (see the ‘Sequence-context-based classification of single-base substitutions’ section). SBSs at cytosine bases in TCN contexts were classified as ‘APOBEC3’, while all other mutations were classified as ‘non-APOBEC3’. Statistical comparisons of the differences in burdens of clustered SBSs and events across various genotypes and cell lines were calculated using two-tailed Mann–Whitney *U-*tests and FDR correction using the Benjamini–Hochberg procedure (Supplementary Table 6).

### Dependency on REV1 in BRCA cell lines

CRISPR dependency data^62,63^ of BRCA cell lines on REV1 was downloaded from DepMapPortal (DepMap 21Q4 Public; https://depmap.org/portal/gene/REV1?tab=overview). The Chronos dependency score is based on data acquired from a cell depletion assay^64^. A lower Chronos score indicates a likelihood that the gene of interest is essential in a given cell line. A score of 0 indicates that a gene is non-essential; correspondingly −1 is comparable to the median of all pan-essential genes. Mutational signature annotation in BRCA cell lines was published previously^4^. BRCA cell lines with a sum of APOBEC3-associated SBS2 and SBS13 of 0 and greater than 80 mutations were considered to be APOBEC-negative and APOBEC-positive, respectively. A total of 27 BRCA cell lines with available Chronos scores and APOBEC-associated mutational signature status allocation were considered in the analysis (Extended Data Fig. 9j and Supplementary Table 7) and the difference in dependency scores on REV1 was compared between two sets of cell lines using one-tailed Mann–Whitney *U*-tests.

### APOBEC3H haplotype I genotyping

APOBEC3H (A3H) haplotype I was genotyped across the relevant SNP loci (rs34522862/rs139292, rs139293, rs139297, rs139298, rs139299, rs139302) using the aligned whole-genome sequencing data and as reported previously^31^. The analysis revealed that BT-474, MDA-MB-453 and JSC-1 cell lines carry A3H haplotype I, whereas JSC-1 and HT-1376 do not.

### Statistical analyses

Statistical comparisons were performed using the tests and corrections indicated in the figure legends.

### Reporting summary

Further information on research design is available in the Nature Research Reporting Summary linked to this paper.

## Online content

Any methods, additional references, Nature Research reporting summaries, source data, extended data, supplementary information, acknowledgements, peer review information; details of author contributions and competing interests; and statements of data and code availability are available at 10.1038/s41586-022-04972-y.

## Supplementary information

Supplementary Information Supplementary Figs. 1–14 and the legends for Supplementary Tables 1–10.

Reporting Summary

Supplementary Table 1

Supplementary Table 2

Supplementary Table 3

Supplementary Table 4

Supplementary Table 5

Supplementary Table 6

Supplementary Table 7

Supplementary Table 8

Supplementary Table 9

Supplementary Table 10

## Data Availability

All sequencing data generated in the study have been deposited in the European Nucleotide Archive database under accession number ERP137590. Access numbers and IDs of sequence files from individual samples are listed in Supplementary Table 1. Source data, including quantification of mutational sequence contexts (Supplementary Table 3) and quantification of mutational signatures (Supplementary Table 4); and SBS, indel and rearrangement mutation calls (Supplementary Tables 9 and 10) are provided. Publicly available source data includes annotation of mutational signatures across human cancer cell lines and human cancers (Fig. 1) accessed from supplementary table 3 of ref. ^4^, and DepMap dependency data of BRCA cell lines on REV1 downloaded from the DepMapPortal (DepMap 21Q4 Public; https://depmap.org/portal/gene/REV1?tab=overview) (Supplementary Table 7).

## Code availability

Software central to analyses in this Article is available online and includes the core computational pipelines (versions listed in Supplementary Table 1) for sequence alignment (https://github.com/cancerit/PCAP-core); identification of SBSs (https://github.com/cancerit/cgpCaVEManWrapper), indels (https://github.com/cancerit/cgpPindel) and rearrangements (https://github.com/cancerit/BRASS); genotyping of specific loci across multiple samples (https://github.com/cancerit/vafCorrect); as well as code for the identification and quantification of SBS sequence contexts (v.1.1; https://github.com/AlexandrovLab/SigProfilerMatrixGenerator); for the quantification of reference sequence contexts (v.1.1.0; https://github.com/cancerit/sequence_utils/releases/tag/1.1.0); for the analysis of mutational signatures (v.1.1.4; https://github.com/AlexandrovLab/SigProfilerExtractor) and for the analysis of clustered mutations (v.1.1.2; https://github.com/AlexandrovLab/SigProfilerSimulator; v.1.1.0 https://github.com/AlexandrovLab/SigProfilerClusters/releases/tag/v1.0.0).
