# Supplementary material for: Mechanisms of APOBEC3 mutagenesis in human cancer cells
Source: Nature. 2022 Jul 20;607(7920):799–807. doi: 10.1038/s41586-022-04972-y (PMC9329121; doi:10.1038/s41586-022-04972-y)

nature portfolio

https://doi.org/10.1038/s41586-022-04972-y

Supplementary information

Mechanisms of APOBEC3 mutagenesis in human cancer cells

In the format provided by the authors and unedited

SI Guide

Mechanisms of APOBEC3 mutagenesis in human cancer cells

Mia Petljak[1*], Alexandra Dananberg[2,8], Kevan Chu[2,8], Erik N. Bergstrom[3,4,5], Josefine Striepen[2], Patrick von Morgen[2], Yanyang Chen[2], Hina Shah[2], Julian E. Sale[6,7], Ludmil B. Alexandrov[3,4,5,] Michael R. Stratton[7*], John Maciejowski[2*]

[1]Broad Institute of MIT and Harvard, Cambridge, MA, 02142, USA.
[2]Molecular Biology Program, Sloan Kettering Institute, Memorial Sloan Kettering Cancer Center, New York, NY, 10065, USA.
[3]Department of Cellular and Molecular Medicine, UC San Diego, La Jolla, CA, 92093, USA.
[4]Department of Bioengineering, UC San Diego, La Jolla, CA, 92093, USA.
[5]Moores Cancer Center, UC San Diego, La Jolla, CA, 92037, USA.
[6]Division of Protein & Nucleic Acid Chemistry, Medical Research Council Laboratory of Molecular Biology, Francis Crick Avenue, Cambridge CB2 0QH, UK.
[7]Cancer, Ageing and Somatic Mutation, Wellcome Sanger Institute, Hinxton, Cambridgeshire, CB10 1SA, UK.
[8]These authors contributed equally.

Keywords: APOBEC3 mutations, kataegis, omikli, mutational signatures, cancer cell lines

*** Corresponding authors:**
John Maciejowski, PhD
Molecular Biology Program
Sloan Kettering Institute
Memorial Sloan Kettering Cancer Center
New York, NY, 10065, USA
maciejoj@mskcc.org

Mia Petljak, PhD
Broad Institute of MIT and Harvard
Cambridge, MA, 02142, USA
mpetljak@broadinstitute.org

Michael R. Stratton, MBBS, PhD
Wellcome Sanger Institute
Cancer, Ageing and Somatic Mutations
Hinxton, Cambridgeshire CB10 1SA, United Kingdom
mrs@sanger.ac.uk

Table of contents:

SI Figure 1 | Uncropped immunoblots from Figure 1c. Proteins were detected by Western blotting using the indicated antibodies. Red rectangles show the cropping location.

SI Figure 2 | Uncropped immunoblots from Figure 3k. Proteins were detected by Western blotting using the indicated antibodies. Red rectangles show the cropping location.

SI Figure 3 | Uncropped immunoblots from Figure 3l. Proteins were detected by Western blotting using the indicated antibodies. Red rectangles show the cropping location.

SI Figure 4 | Uncropped agarose gels from Extended Data Fig. 1b. DNA was stained with ethidium bromide. Red rectangles show the cropping location.

SI Figure 5 | Uncropped immunoblots from Extended Data Fig. 1g. Proteins were detected by Western blotting using the indicated antibodies. Red rectangles show the cropping location.

SI Figure 6 | Uncropped immunoblots from Extended Data Fig. 1h-k. Proteins were detected by Western blotting using the indicated antibodies. Red rectangles show the cropping location.

SI Figure 7 | Uncropped immunoblots from Extended Data Fig. 1l-m. Proteins were detected by Western blotting using the indicated antibodies. Red rectangles show the cropping location.

SI Figure 8 | Uncropped immunoblots from Extended Data Fig. 3j. Proteins were detected by Western blotting using the indicated antibodies. Red rectangles show the cropping location.

SI Figure 9 | Uncropped immunoblots from Extended Data Fig. 7b-c. Proteins were detected by Western blotting using the indicated antibodies. Red rectangles show the cropping location.

SI Figure 10 | Uncropped immunoblots from Extended Data Fig. 7d. Proteins were detected by Western blotting using the indicated antibodies. Red rectangles show the cropping location.

SI Figure 11 | Uncropped immunoblots from Extended Data Fig. 7e-f. Proteins were detected by Western blotting using the indicated antibodies. Red rectangles show the cropping location.

SI Figure 12 | Uncropped immunoblots from Extended Data Fig. 7g. Proteins were detected by Western blotting using the indicated antibodies. Red rectangles show the cropping location.

SI Figure 13 | Uncropped immunoblots from Extended Data Fig. 7h. Proteins were detected by Western blotting using the indicated antibodies. Red rectangles show the cropping location.

SI Figure 14 | Uncropped immunoblots from Extended Data Fig. 9a. Proteins were detected by Western blotting using the indicated antibodies. Red rectangles show the cropping location.

SI Table 1 | Sample overview, sequence file access numbers, sequencing and mutation calling, and downloaded data from Figure 1.

SI Table 2 | Mutation filtering summary.

SI Table 3 | Mutation matrices.

SI Table 4 | Mutational signature analyses.

SI Table 5 | List of key reagents and resources.

SI Table 6 | Clustered mutations overview.

SI Table 7 | CRISPR dependency data of BRCA cell lines on REV1.

SI Table 8 | Single base substitution calls.

SI Table 9 | Indel calls.

SI Table 10 | Rearrangement calls.

SI Figure 1. Petljak et al.

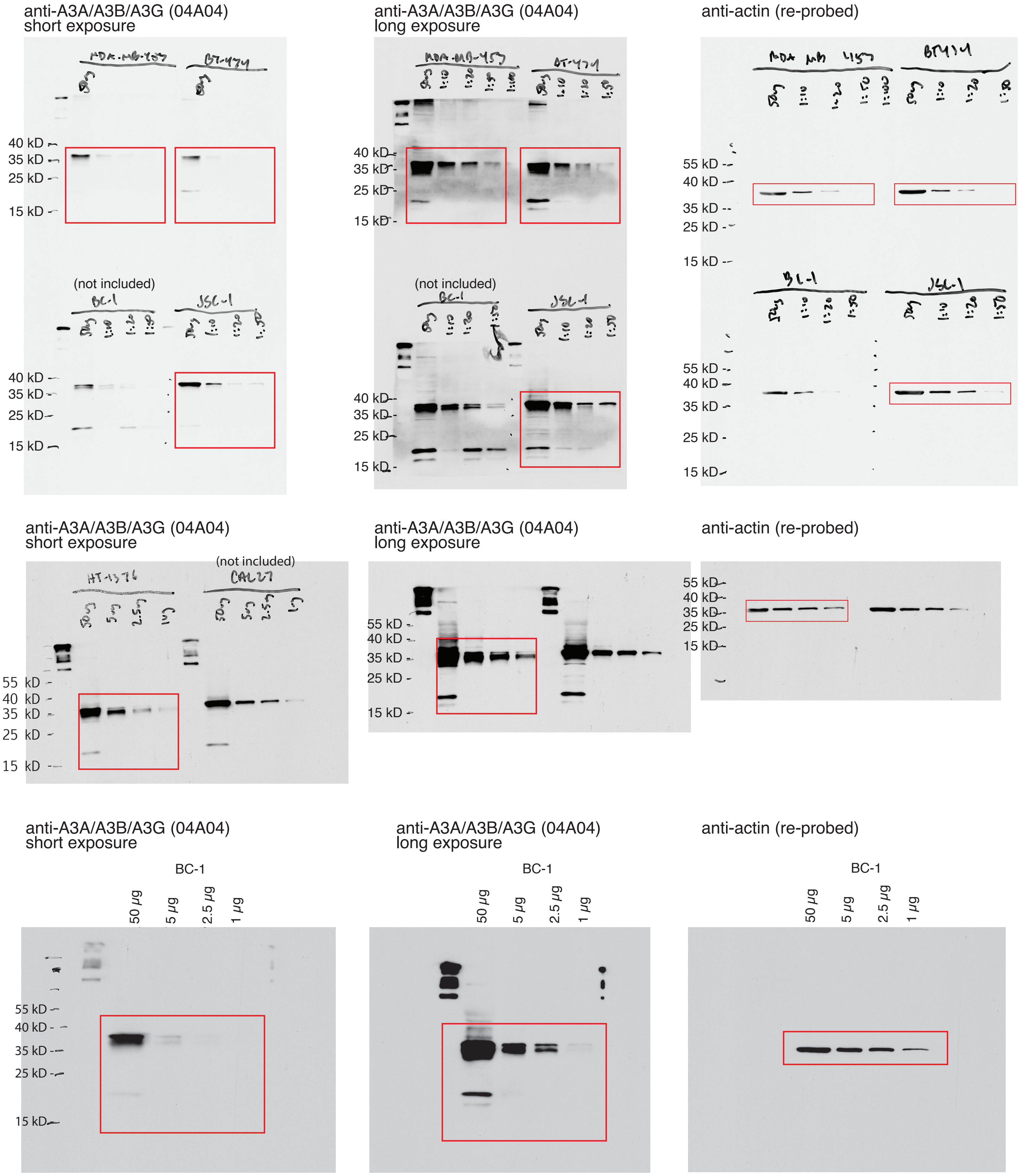

SI Figure 2. Petljak et al.

anti-A3A (01D05)

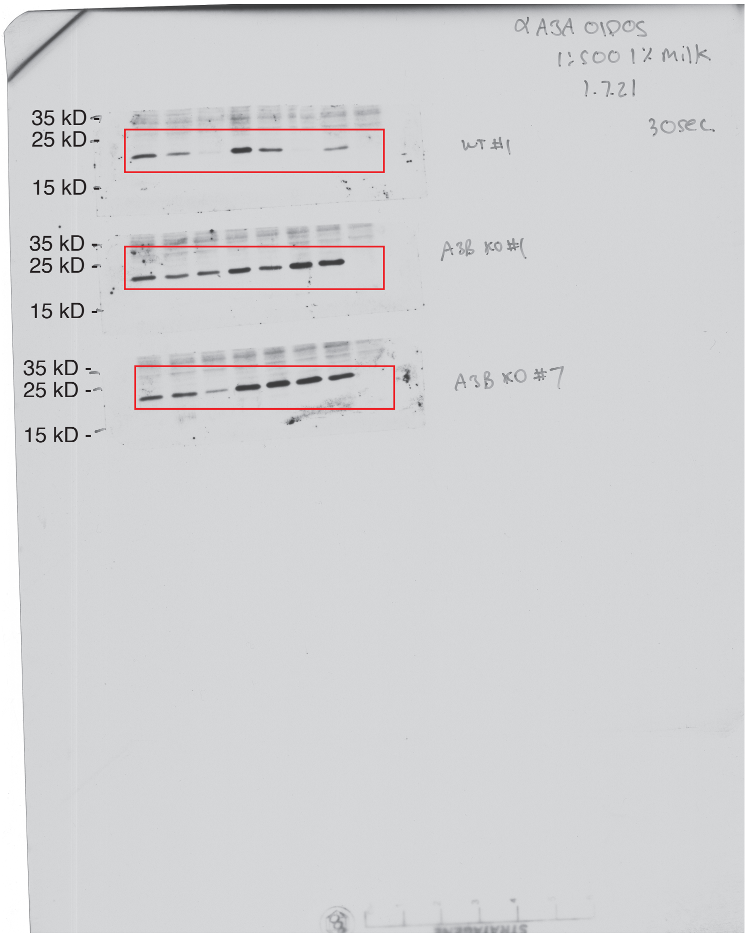

anti-actin (re-probed)

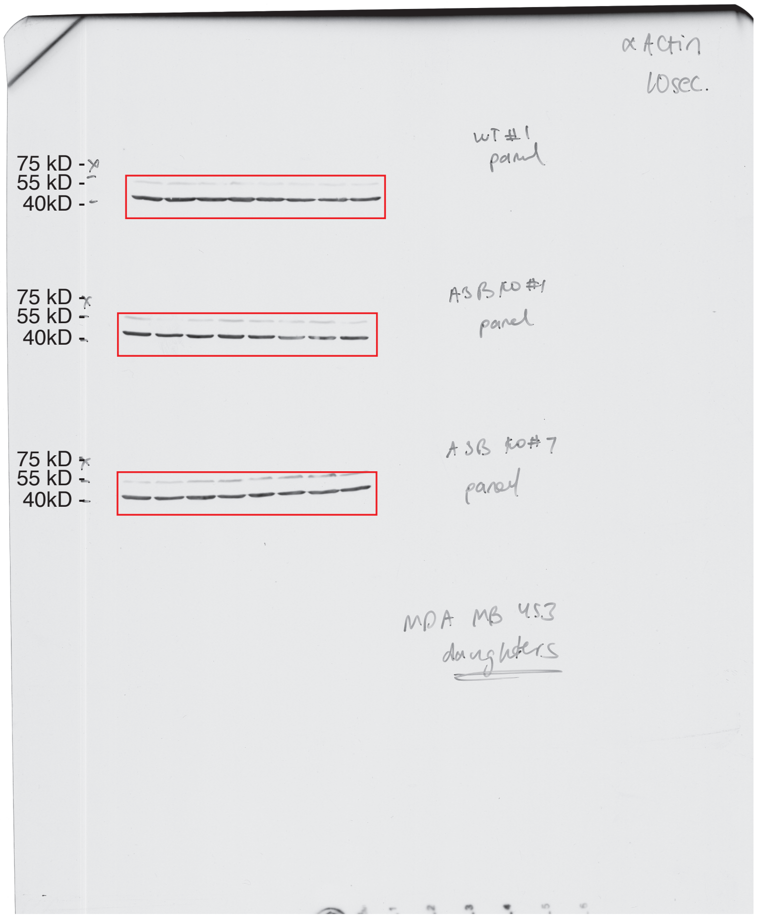

SI Figure 3. Petljak et al.

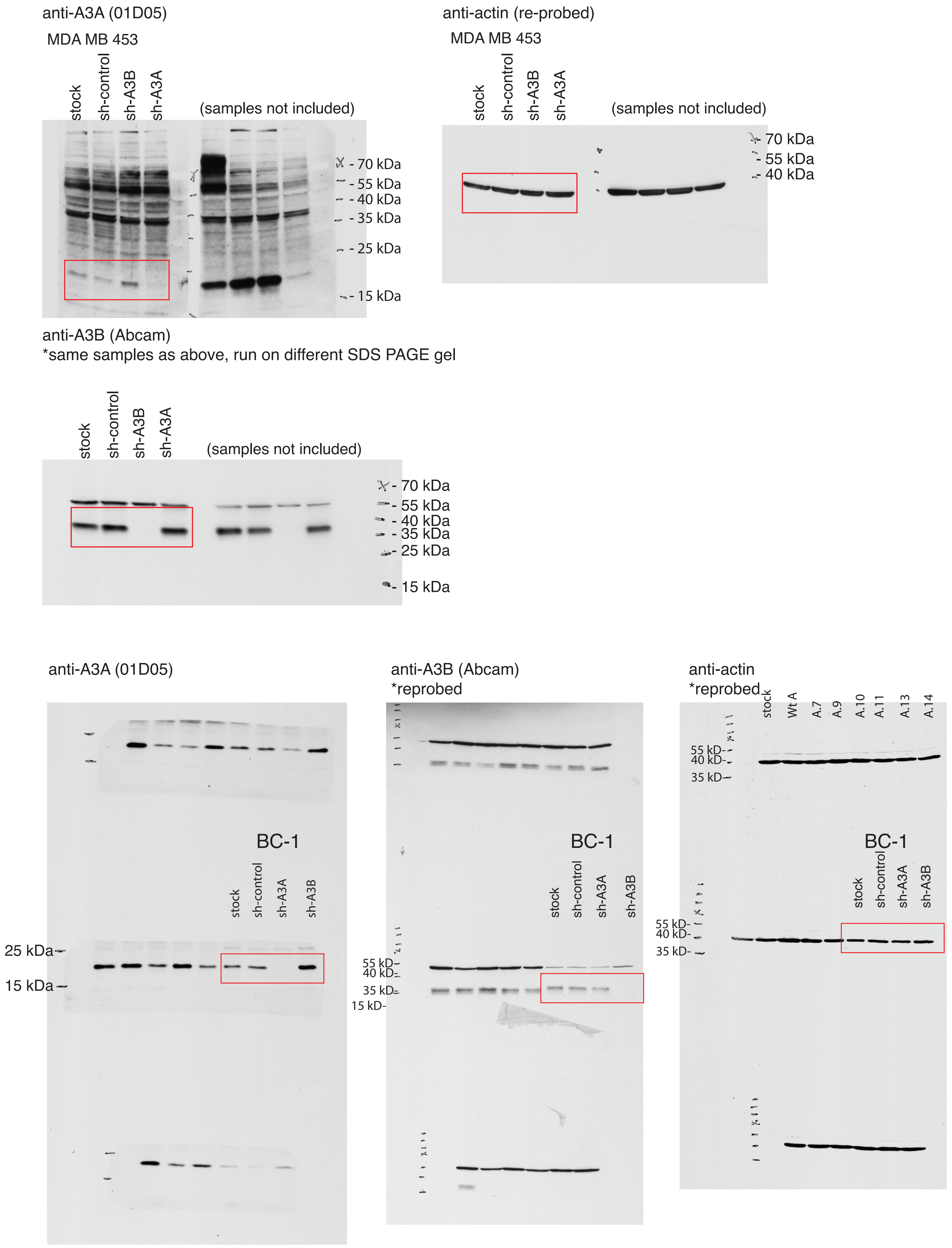

SI Figure 4. Petljak et al.

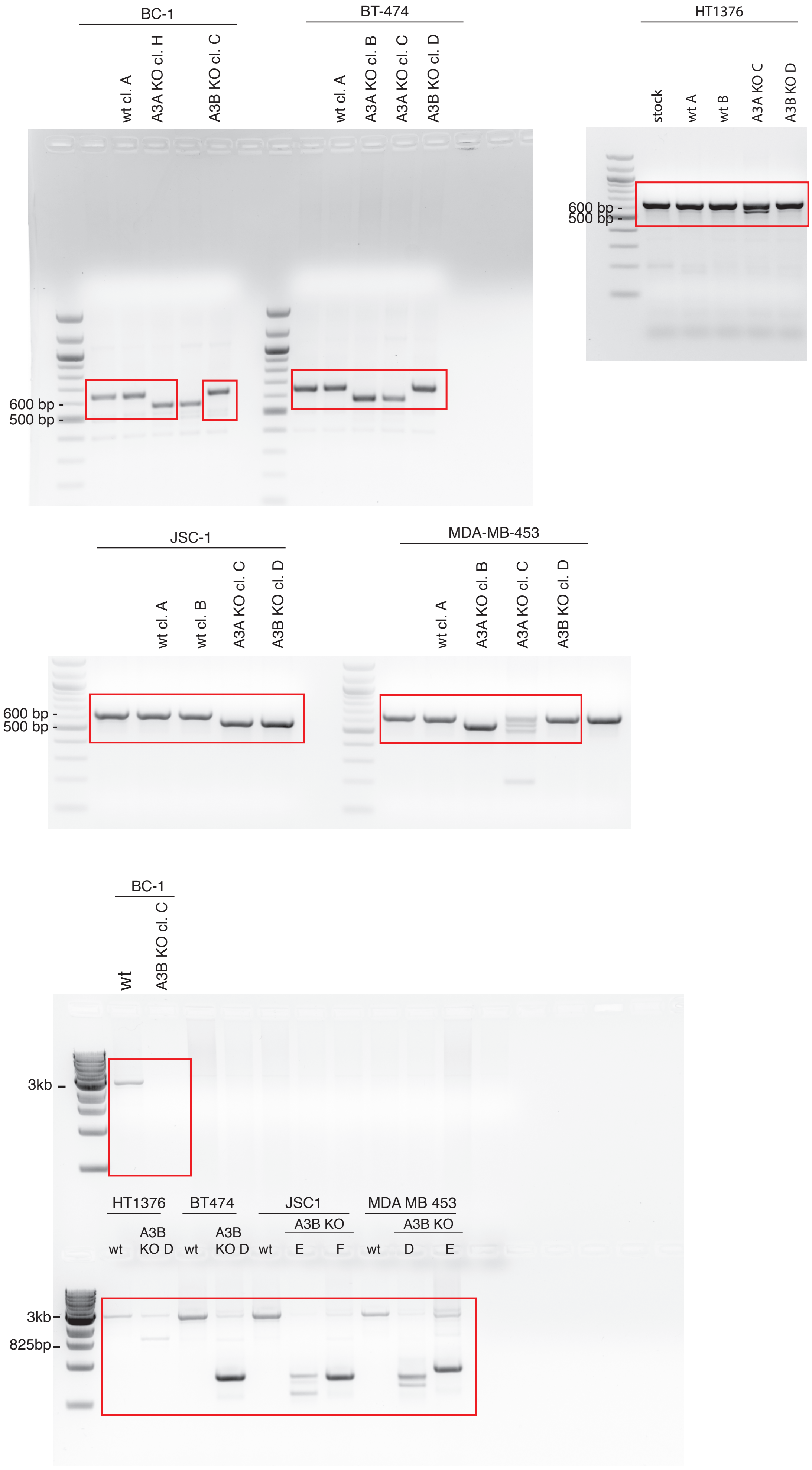

SI Figure 5. Petljak et al.

anti-A3A (01D05)

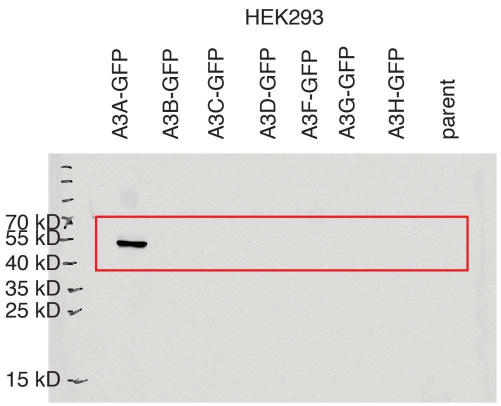

anti-A3A/B/G (04A04)

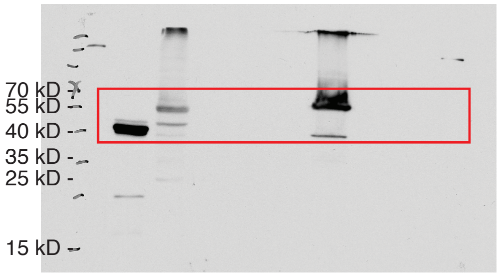

anti-GFP

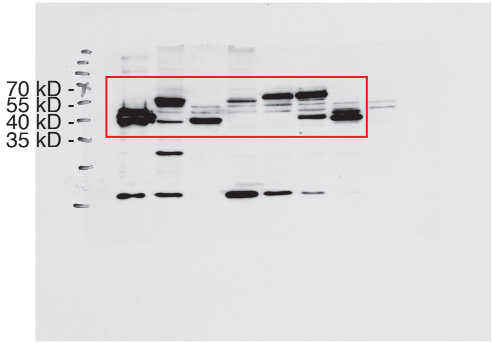

SI Figure 6. Petljak et al.

Related to Extended Data Fig. 1h

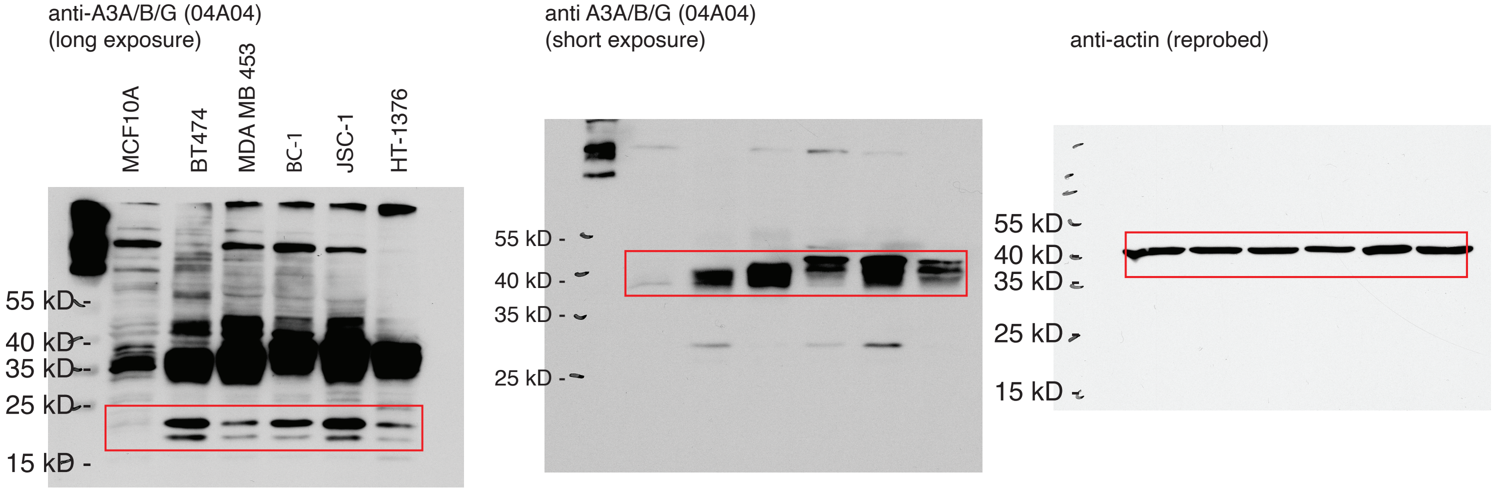

Related to Extended Data Fig. 1i

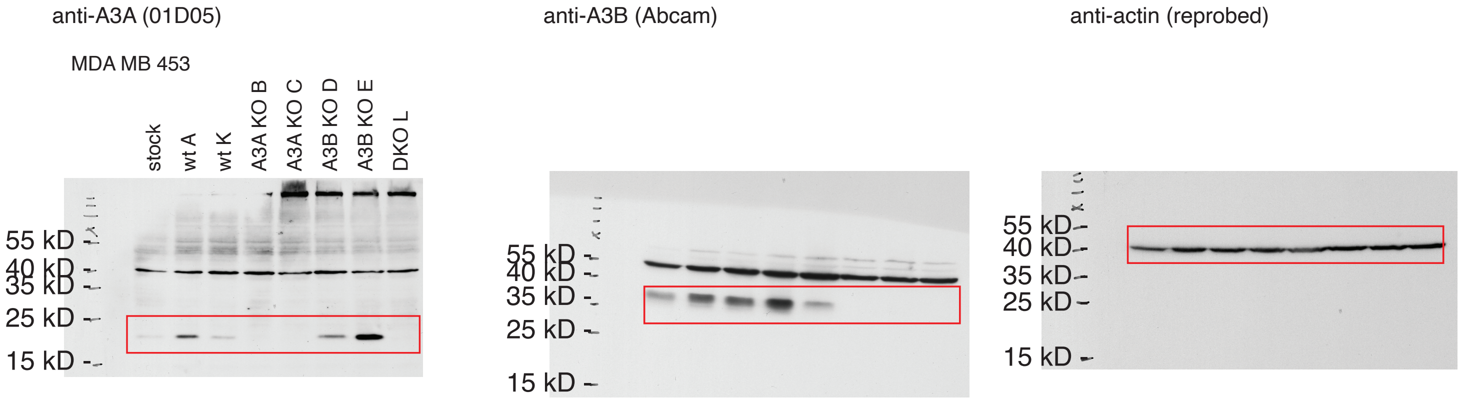

Related to Extended Data Fig. 1j

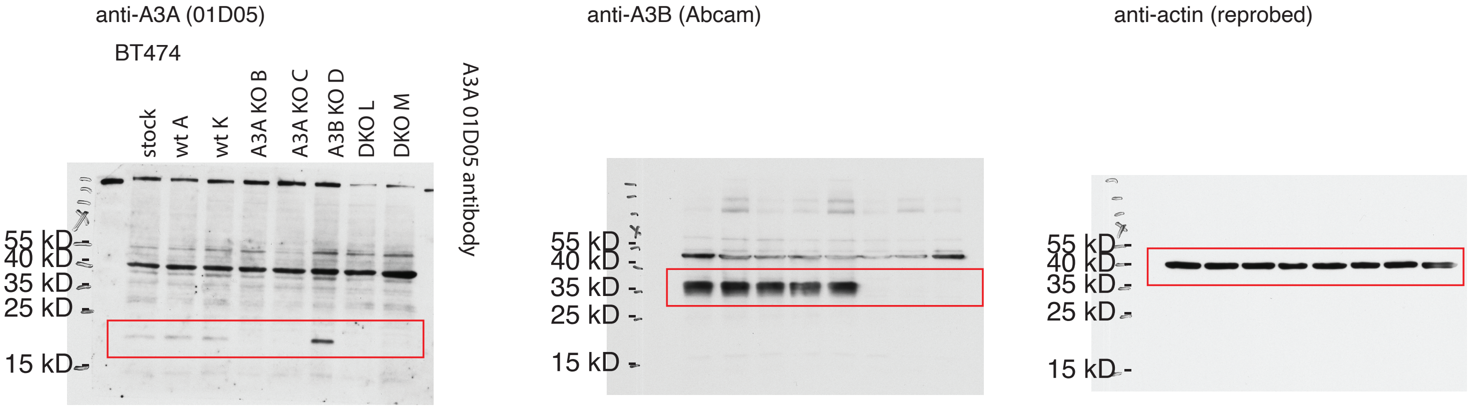

Related to Extended Data Fig. 1k

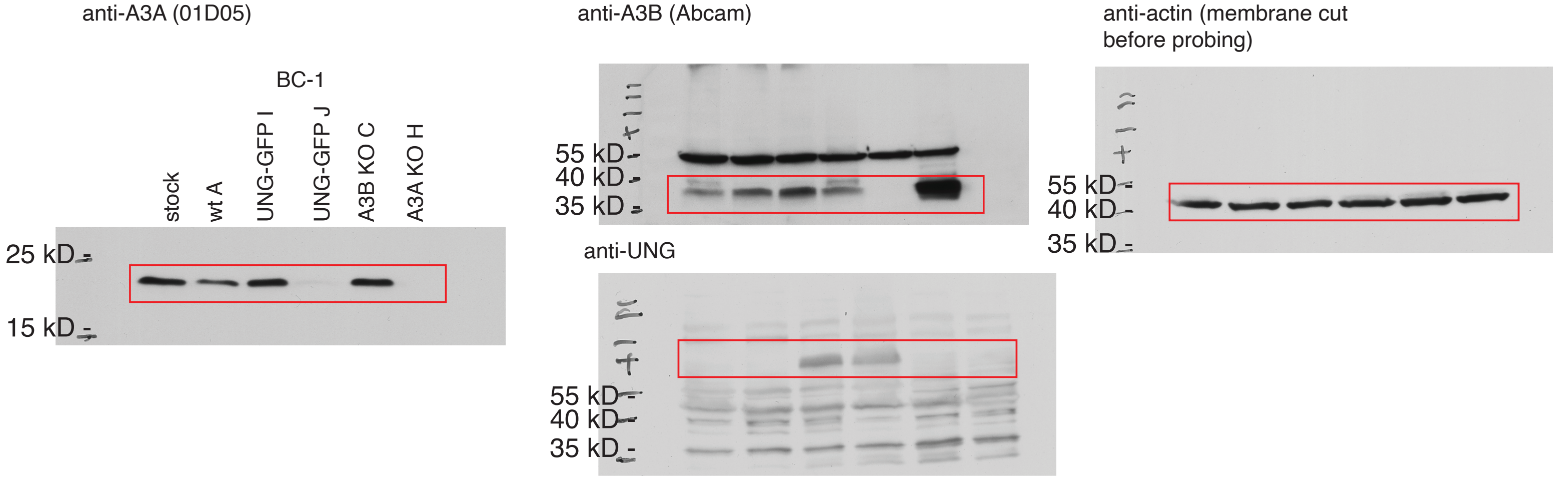

SI Figure 7. Petljak et al.

Related to Extended Data Fig. 1l

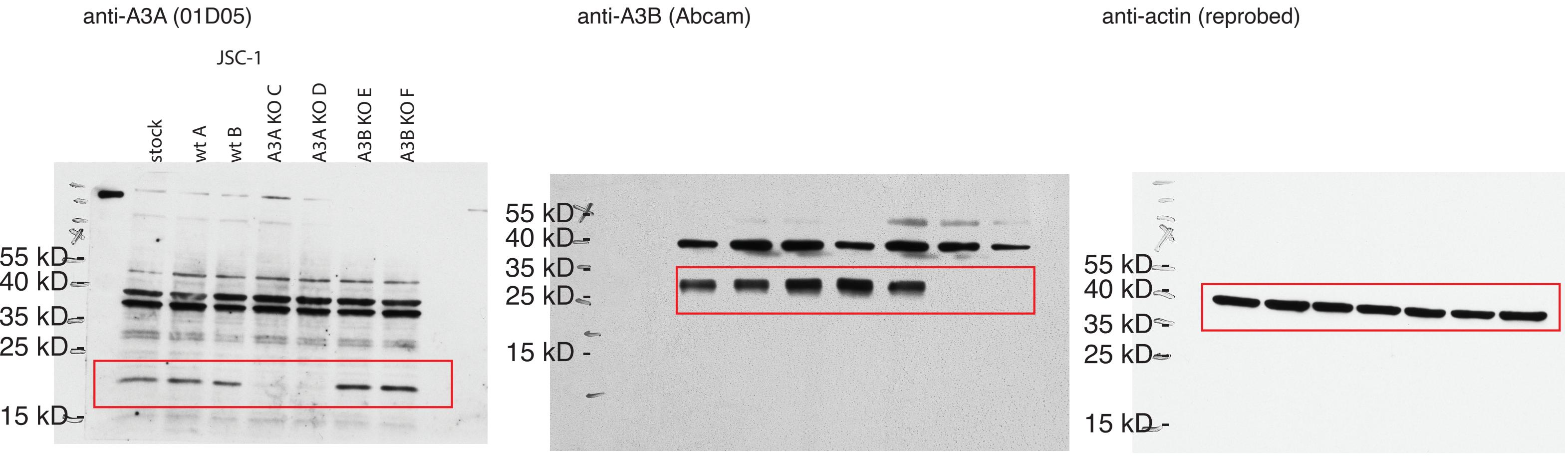

Related to Extended Data Fig. 1m

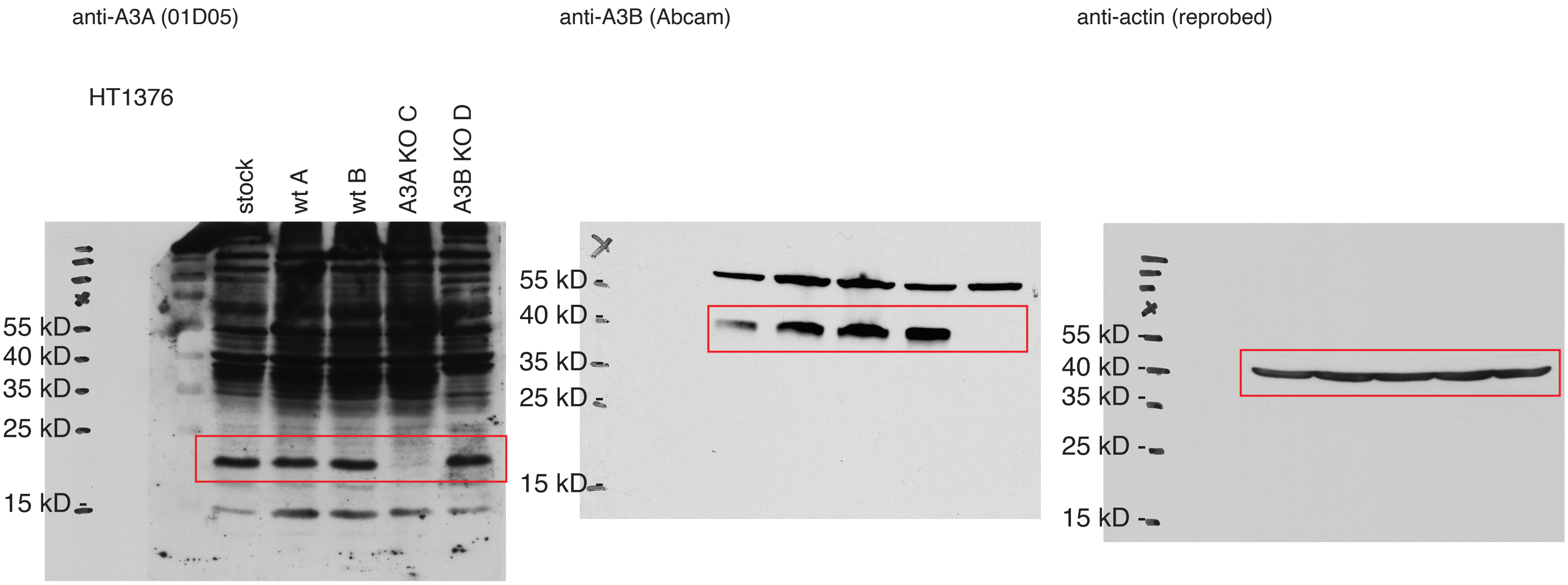

SI Figure 8. Petljak et al.

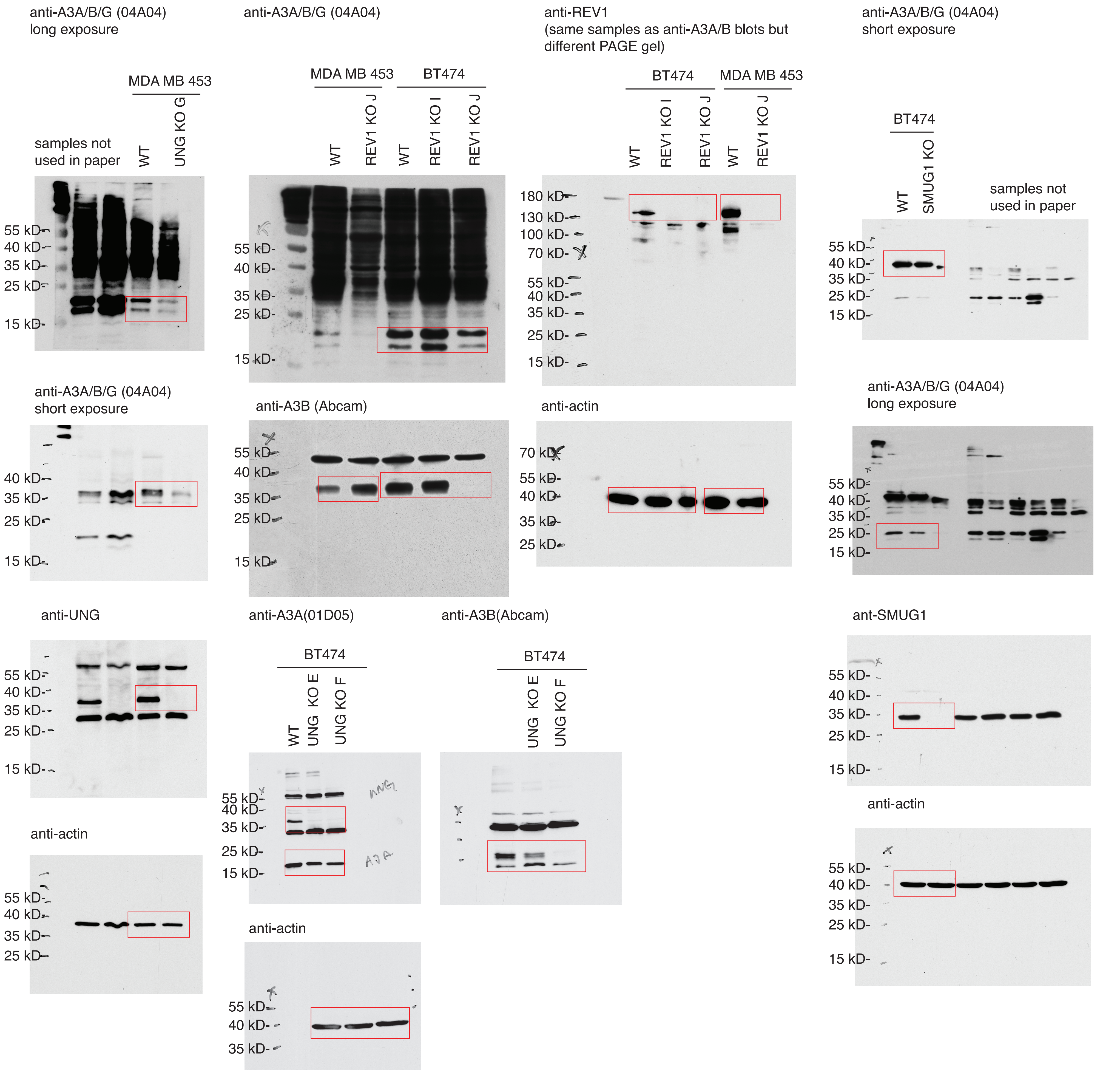

SI Figure 9. Petljak et al.

Related to Extended Data Fig. 7b

anti-A3B (Abcam)

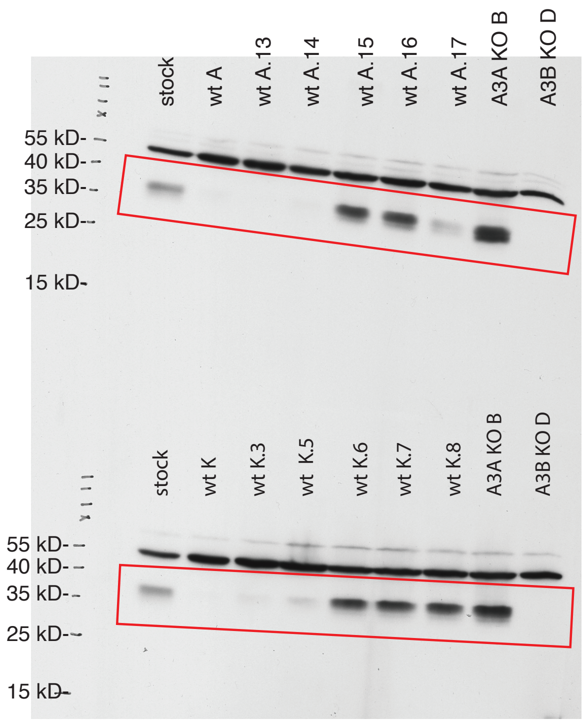

anti-actin
(stripped and reprobed)

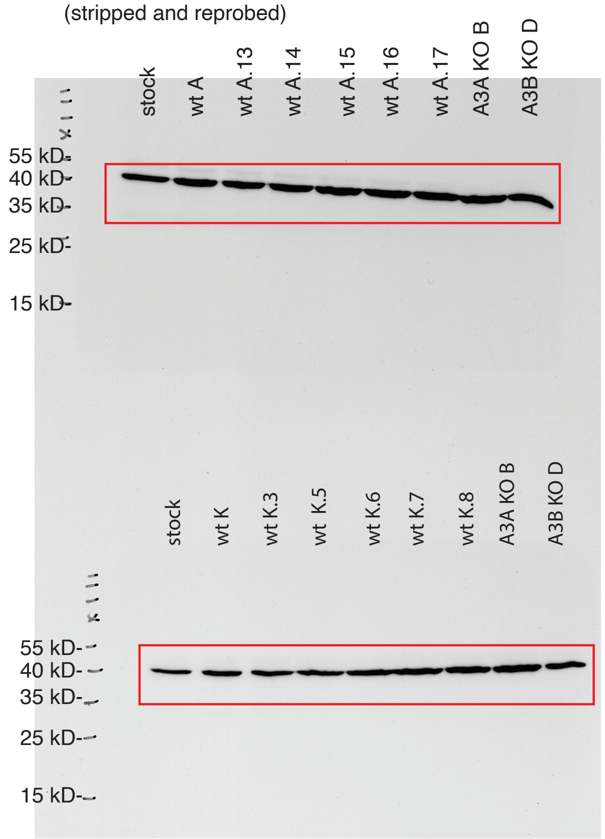

Related to Extended Data Fig. 7c

anti-A3A (01D05)

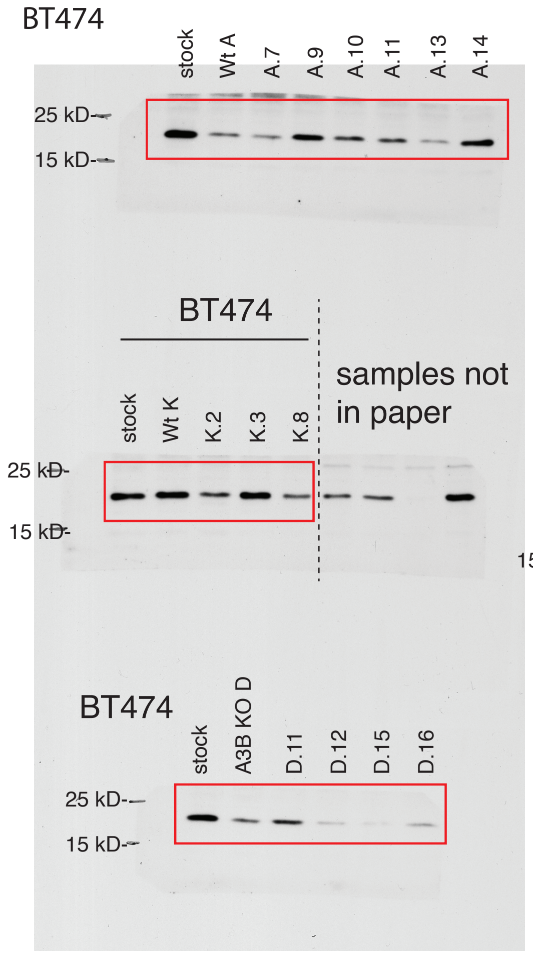

anti-A3B (Abcam)

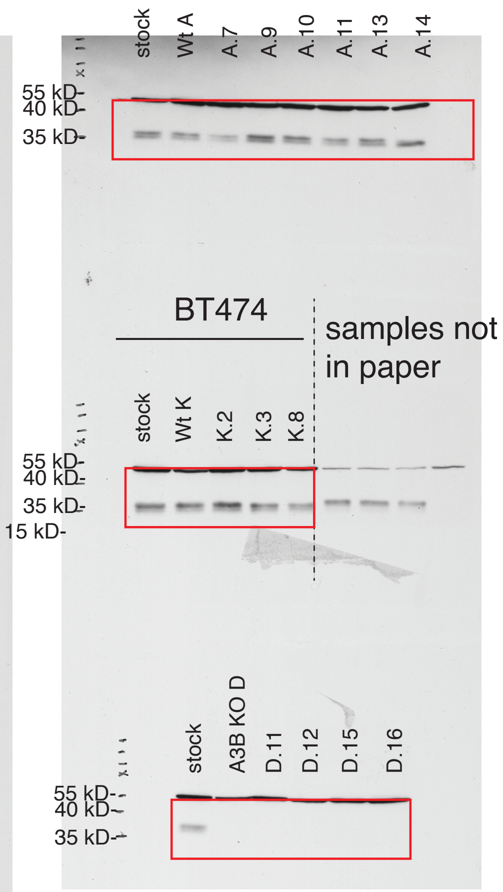

anti-actin

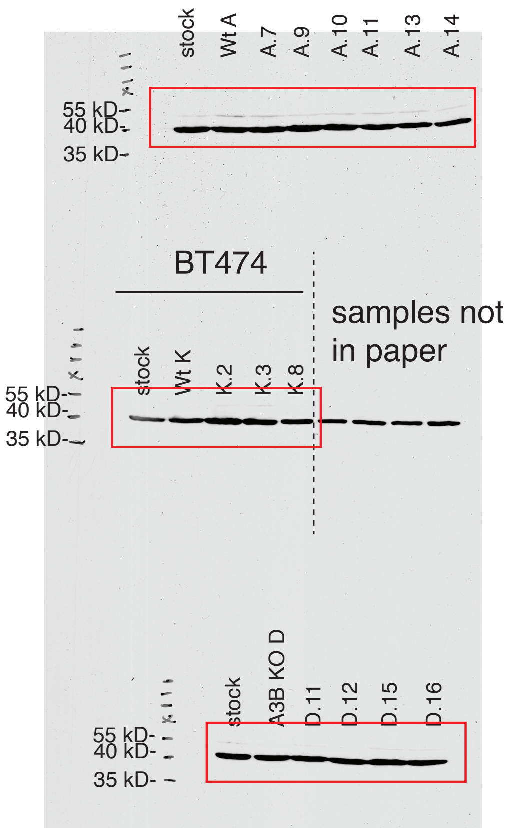

SI Figure 10. Petljak et al.

anti-A3A (01D05)

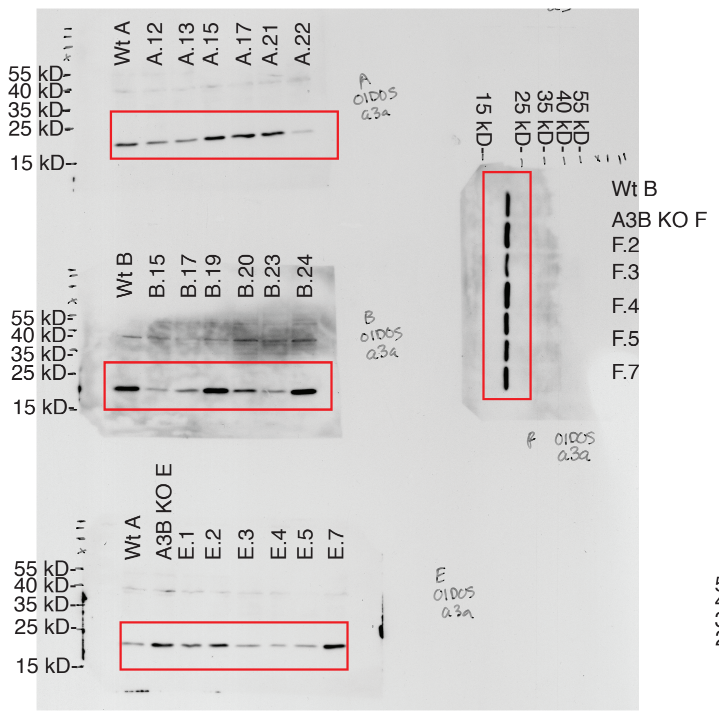

anti-A3B (Abcam)
(same samples as anti-A3A, anti-actin blots but different PAGE gel)

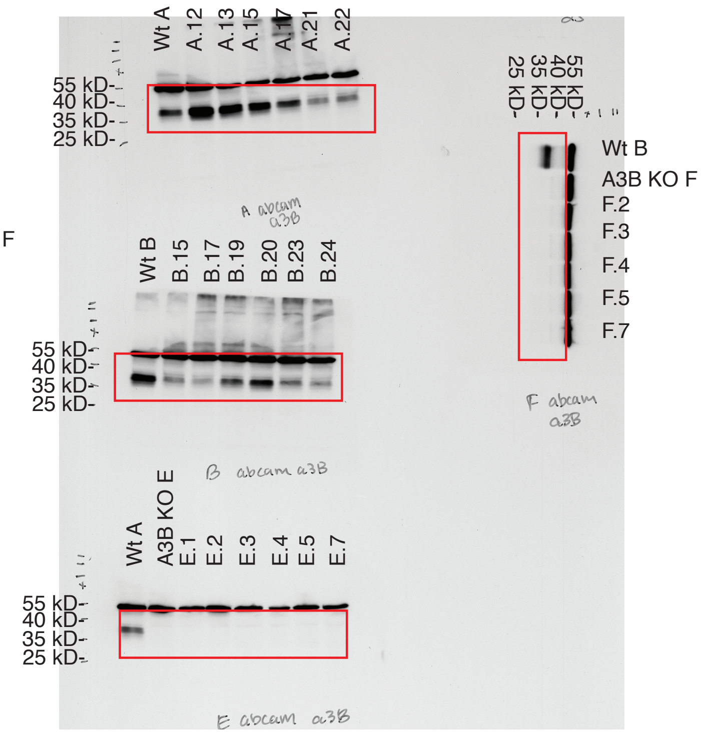

anti-actin
(reprobed after anti-A3A)

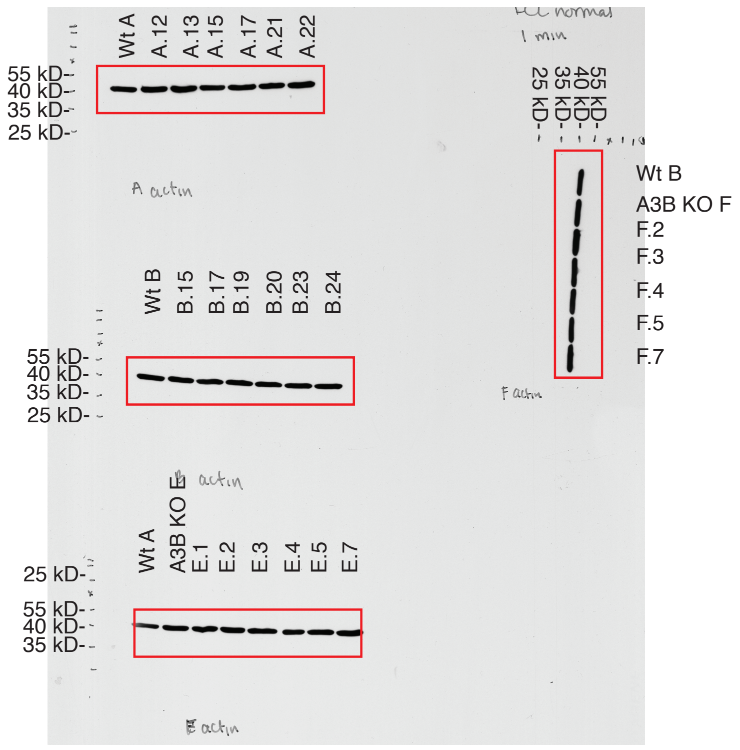

SI Figure 11. Petljak et al.

Related to Extended Data Fig. 7e

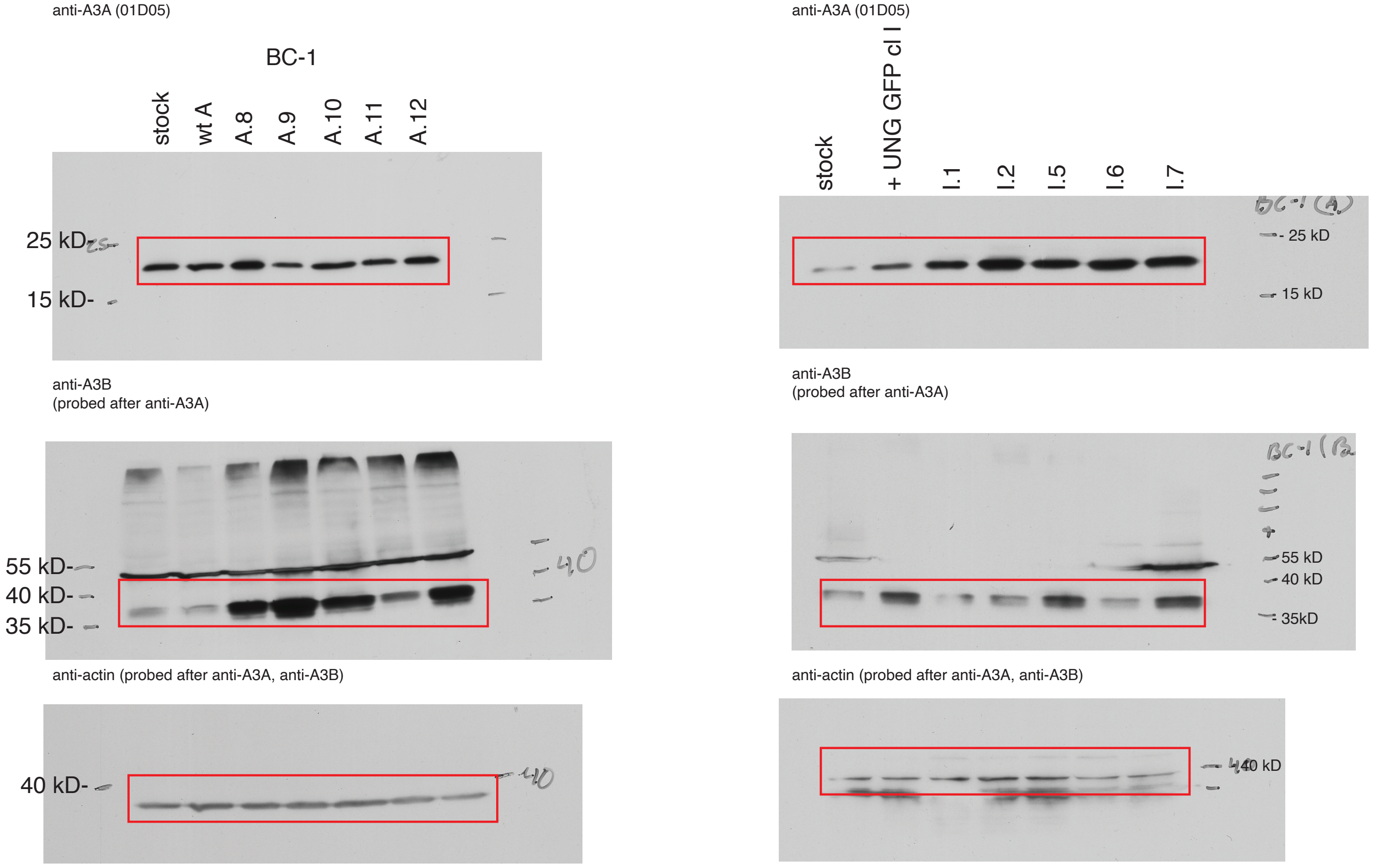

Related to Extended Data Fig. 7f

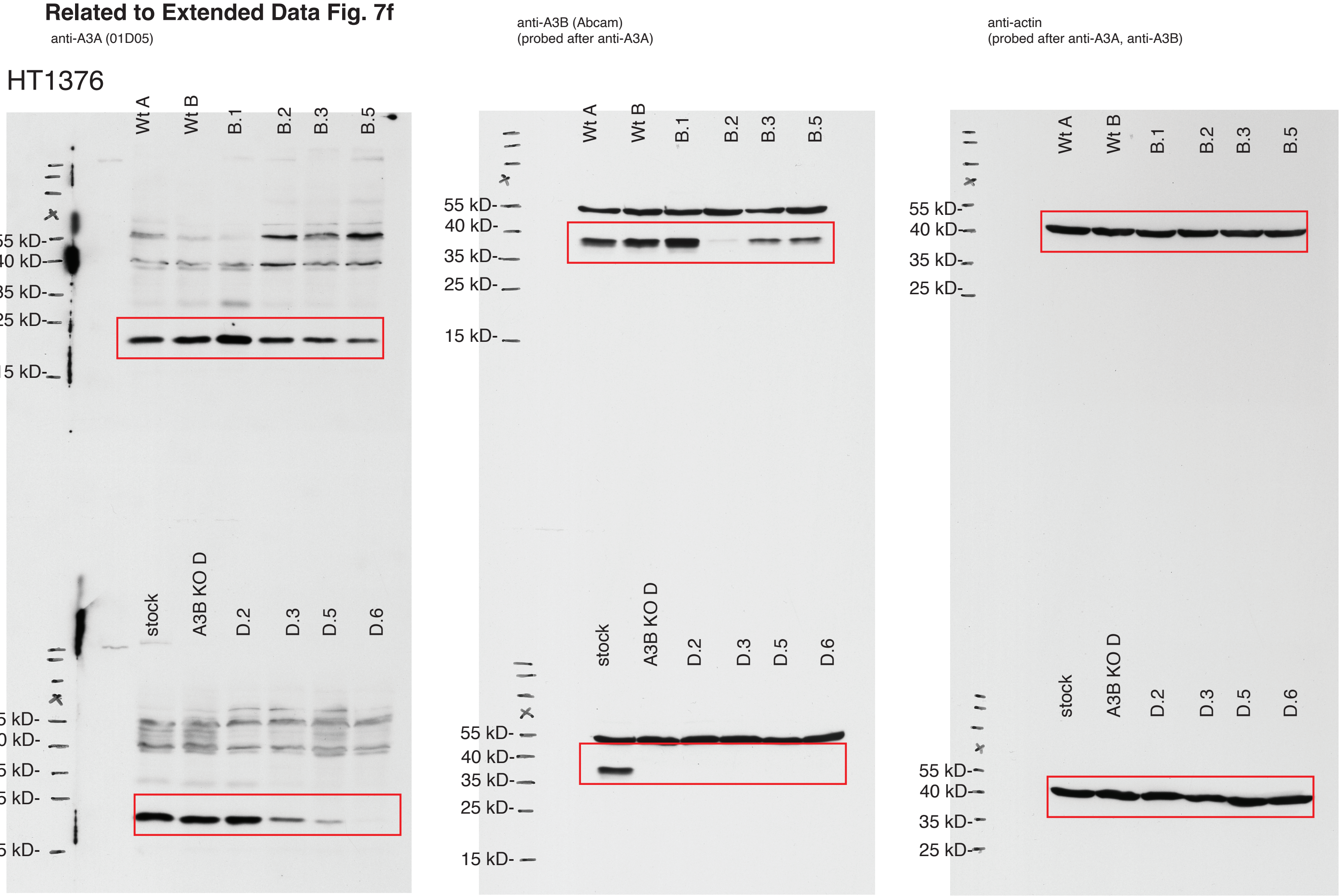

SI Figure 12. Petljak et al.

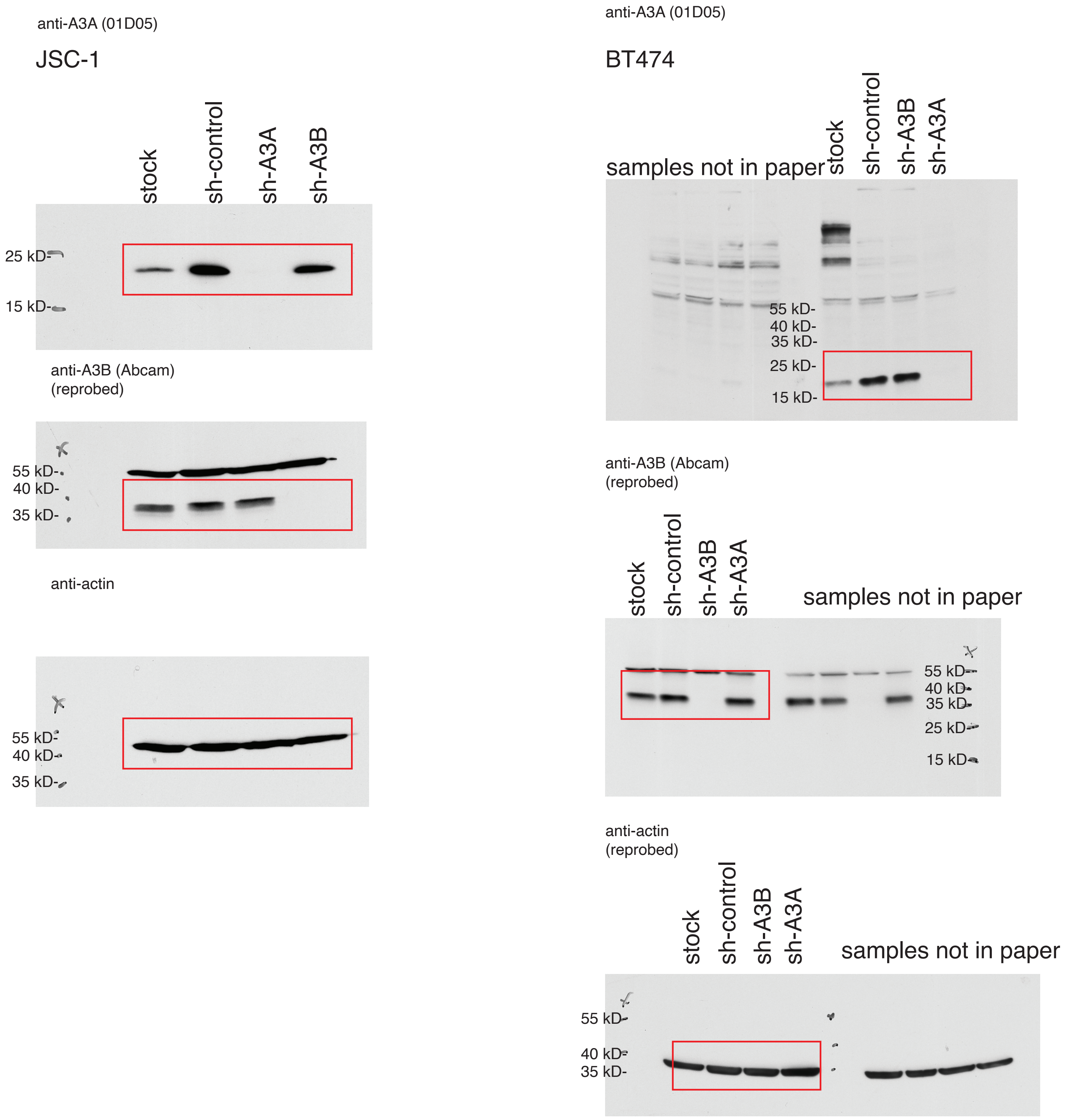

SI Figure 13. Petljak et al.

top: anti-A3A (01D05)

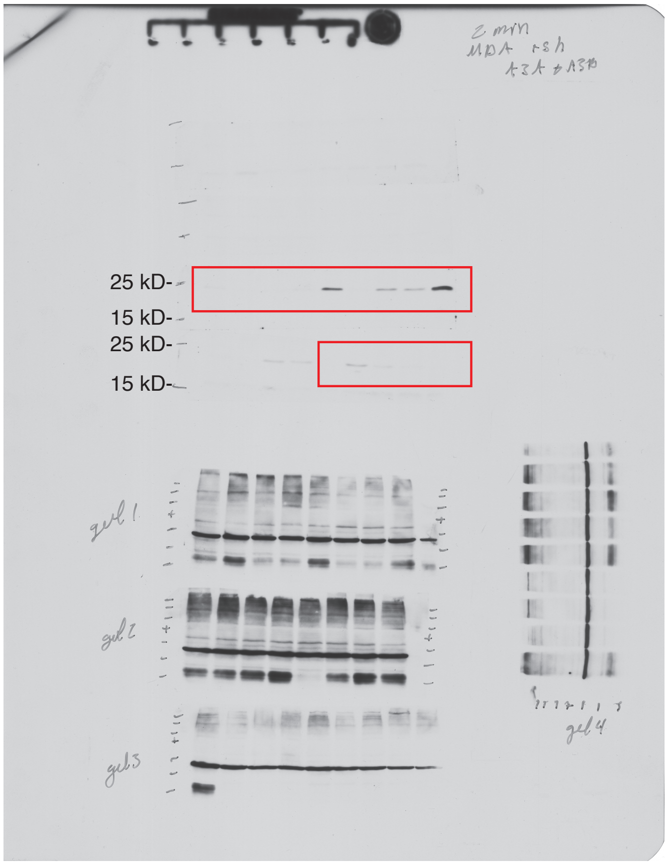

anti-actin

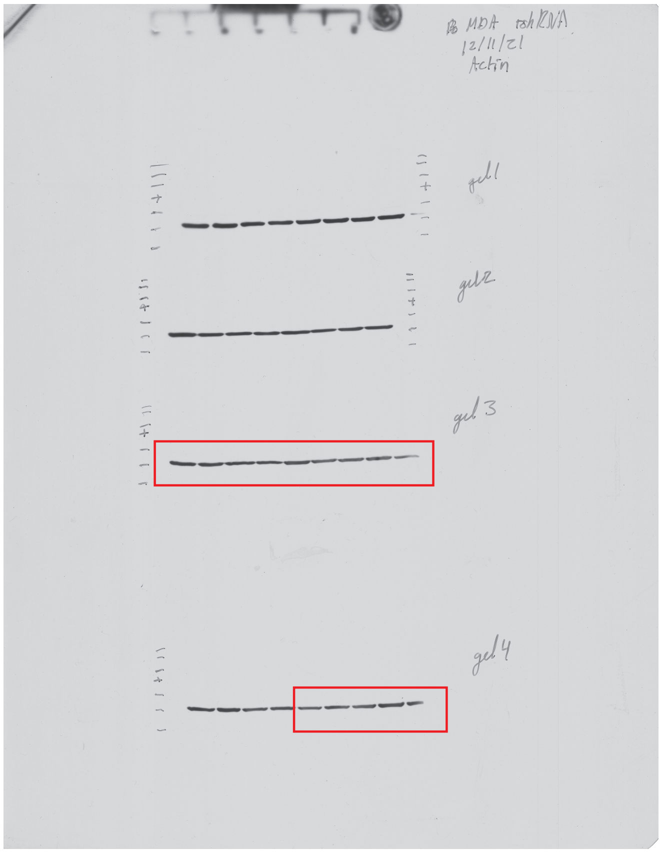

bottom: anti-A3B (Abcam)

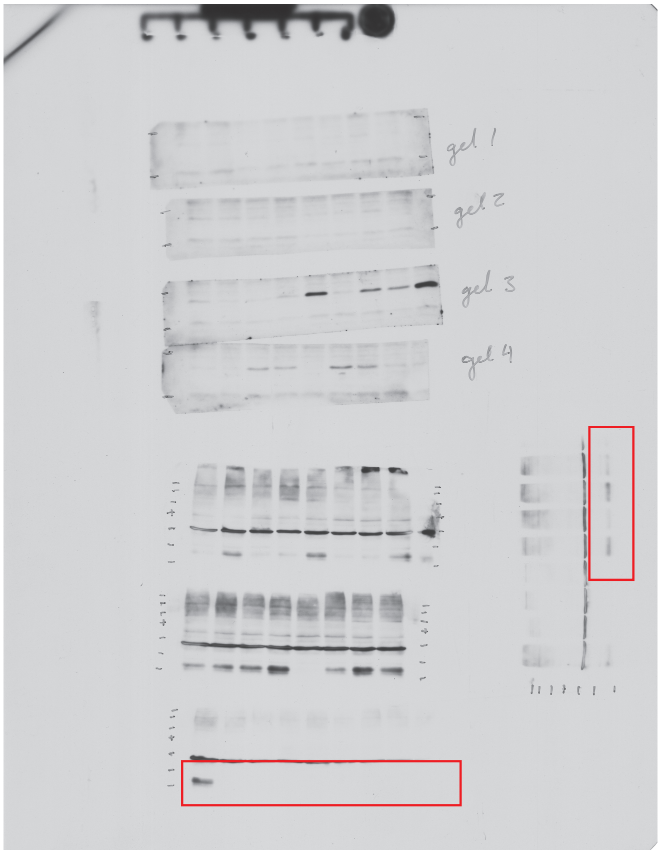

SI Figure 14. Petljak et al.

anti-A3A/B/G (04A04)

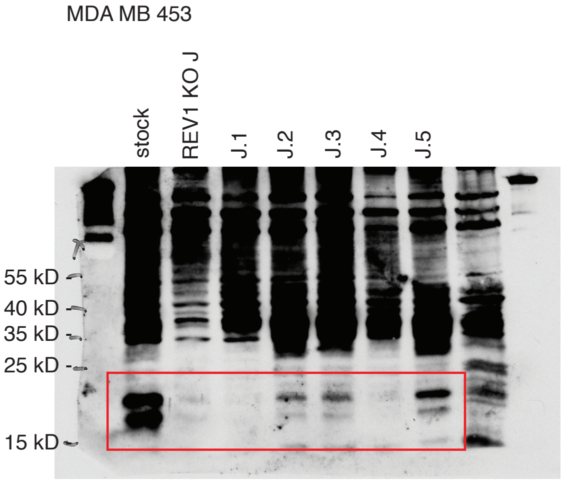

anti-actin (reprobed)

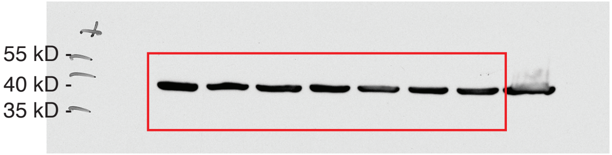

Supplement: Supplementary file 1 — Supplementary Figs. 1–14 and the legends for Supplementary Tables 1–10. [file 41586_2022_4972_MOESM1_ESM.pdf]
