# Supplementary material for: Mechanisms of APOBEC3 mutagenesis in human cancer cells
Source: Nature. 2022 Jul 20;607(7920):799–807. doi: 10.1038/s41586-022-04972-y (PMC9329121; doi:10.1038/s41586-022-04972-y)
Supplement: Supplementary file 2 — Reporting Summary [file 41586_2022_4972_MOESM2_ESM.pdf]

nature portfolio

Corresponding author(s): John Maciejowski

Last updated by author(s): May 24, 2022

Reporting Summary

Nature Portfolio wishes to improve the reproducibility of the work that we publish. This form provides structure for consistency and transparency in reporting. For further information on Nature Portfolio policies, see our Editorial Policies and the Editorial Policy Checklist.

Statistics

For all statistical analyses, confirm that the following items are present in the figure legend, table legend, main text, or Methods section.

n/a	Confirmed	
☐	☒	The exact sample size (*n*) for each experimental group/condition, given as a discrete number and unit of measurement
☐	☒	A statement on whether measurements were taken from distinct samples or whether the same sample was measured repeatedly
☐	☒	The statistical test(s) used AND whether they are one- or two-sided<br>*Only common tests should be described solely by name; describe more complex techniques in the Methods section.*
☒	☐	A description of all covariates tested
☐	☒	A description of any assumptions or corrections, such as tests of normality and adjustment for multiple comparisons
☐	☒	A full description of the statistical parameters including central tendency (e.g. means) or other basic estimates (e.g. regression coefficient) AND variation (e.g. standard deviation) or associated estimates of uncertainty (e.g. confidence intervals)
☐	☒	For null hypothesis testing, the test statistic (e.g. *F*, *t*, *r*) with confidence intervals, effect sizes, degrees of freedom and *P* value noted<br>*Give P values as exact values whenever suitable.*
☒	☐	For Bayesian analysis, information on the choice of priors and Markov chain Monte Carlo settings
☒	☐	For hierarchical and complex designs, identification of the appropriate level for tests and full reporting of outcomes
☒	☐	Estimates of effect sizes (e.g. Cohen's *d*, Pearson's *r*), indicating how they were calculated

Our web collection on statistics for biologists contains articles on many of the points above.

Software and code

Policy information about availability of computer code

Data collection	Indirect immunofluorescence microscopy was performed using softWoRx v7.0.0 and CentOS v6.3 operating system. Agarose gels and Western blots were imaged using the ChemiDoc MP Imaging system (BioRad). Cell growth was analyzed using IncuCyte v2020b. Real Time quantitative PCR data were collected using QuantStudio Real-Time PCR software v1.3. Deaminase assays were imaged using ImageStudio (Licor). RNA editing assays were performed using QuantSoft analysis software (BioRad).
Data analysis	Indirect immunofluorescence microscopy data and deaminase assays were analyzed using Fiji v1.53b. qPCR data were analyzed using QuantStudio Real-Time PCR software v1.3. Data were plotted and statistical analyses were performed using Graphpad Prism v9.3.0 (Graphpad Software Inc.). RNA editing assays were analyzed using QuantSoft analysis software (BioRad). Sanger sequencing alignments and knockout validation were performed using Benchling [Biology Software] 2022 (https://benchling.com). Sequencing reads were aligned to the reference human genome (GRCh37) using Burrows-Wheeler Alignment (BWA)-MEM (https://github.com/cancerit/PCAP-core). Somatic single base substitutions (SBS) were discovered with CaVEMan (https://github.com/cancerit/cgpCaVEManWrapper). Rearrangements and indels were identified with BRASS (https://github.com/cancerit/BRASS) and cgpPindel57 (https://github.com/cancerit/cgpPindel). SigProfilerMatrixGenerator (v.1.1; https://github.com/AlexandrovLab/SigProfilerMatrixGenerator) was used to categorize SBSs into three separate sequence-context based classifications. Trinucleotide and pentanucleotide sequence motifs were quantified with sequence_utils (v.1.1.0, https://github.com/cancerit/sequence_utils/releases/tag/1.1.0;https://github.com/cancerit/sequence_utils/wiki#sequence-context-of-regions-processed-by-caveman). Mutational signature analyses were performed using the SigProfilerExtractor tool (v. 1.1.4; https://github.com/AlexandrovLab/SigProfilerExtractor). Clustered mutations were analyzed using SigProfilerClusters (v1.0.0; https://github.com/AlexandrovLab/SigProfilerClusters/releases/tag/v1.0.0). Figures assembled using Illustrator 2020 and Photoshop 2020 (Adobe).

For manuscripts utilizing custom algorithms or software that are central to the research but not yet described in published literature, software must be made available to editors and reviewers. We strongly encourage code deposition in a community repository (e.g. GitHub). See the Nature Portfolio guidelines for submitting code & software for further information.

Data

Policy information about availability of data

All manuscripts must include a data availability statement. This statement should provide the following information, where applicable:

- Accession codes, unique identifiers, or web links for publicly available datasets
- A description of any restrictions on data availability
- For clinical datasets or third party data, please ensure that the statement adheres to our policy

All sequencing data generated in the study have been deposited in the European Nucleotide Archive database with the accession number ERP137590. Access numbers and IDs of sequence files from individual samples are listed in Supplementary Table 1. Source data, including quantification of mutational sequence contexts (Supplementary Table 3) and quantification of mutational signatures (Supplementary Table 4); SBS, indel and rearrangement mutation calls (Supplementary Tables 9-10) are provided. Publicly available source data includes annotation of mutational signatures across human cancer cell lines and human cancers (Fig. 1) accessed from Supplementary Table 3 of previously published work (Petljak, M. et al. Characterizing Mutational Signatures in Human Cancer Cell Lines Reveals Episodic APOBEC Mutagenesis. Cell 176, 1282–1294.e20 (2019).); and DepMap dependency data of BRCA cell lines on REV1 downloaded from DepMapPortal (DepMap 21Q4 Public; https://depmap.org/portal/gene/REV1?tab=overview) (Supplementary Table 7).

Field-specific reporting

Please select the one below that is the best fit for your research. If you are not sure, read the appropriate sections before making your selection.

☒ Life sciences ☐ Behavioural & social sciences ☐ Ecological, evolutionary & environmental sciences

For a reference copy of the document with all sections, see nature.com/documents/nr-reporting-summary-flat.pdf

Life sciences study design

All studies must disclose on these points even when the disclosure is negative.

Sample size	Statistical methods were not used to pre-determine sample size. The sample size follows common standards (n = 3 or more biological replicates) and<br>similar publications in the field. For examples see Jalili et al, 2020 June 12; DOI: 10.1038/s41467-020-16802-8; Cortez et al, 2019 Dec 16; DOI: 10.1371/journal.pgen.1008545 or list of references in the main text. Sample size is reported in legends for main and Extended figures.
Data exclusions	Some cell line daughter clones were excluded from statistical testing because they are suspected to be polyclonal. These are clearly marked in red font throughout figures and in associated legends.
Replication	All attempts at replication were successful. The number of experiments performed are indicated in the figures and legends.
Randomization	All the samples analyzed in this study were clearly recognizable. i.e. KO cell lines can be identified by Western blotting. For this reason randomization was not performed and findings are supported by independent biological replicates.
Blinding	For the same reasons as randomization, and the inclusion of appropriate negative and positive controls, blinding was not required or not possible due to the experimental setup. Analyses that could be biased (i.e. H2AX foci counting) were performed using dedicated software.

Reporting for specific materials, systems and methods

We require information from authors about some types of materials, experimental systems and methods used in many studies. Here, indicate whether each material, system or method listed is relevant to your study. If you are not sure if a list item applies to your research, read the appropriate section before selecting a response.

Materials & experimental systems

n/a	Involved in the study	
☐	☒	Antibodies
☐	☒	Eukaryotic cell lines
☒	☐	Palaeontology and archaeology
☒	☐	Animals and other organisms
☒	☐	Human research participants
☒	☐	Clinical data
☒	☐	Dual use research of concern

Methods

n/a	Involved in the study	
☒	☐	ChIP-seq
☒	☐	Flow cytometry
☒	☐	MRI-based neuroimaging

Antibodies

Antibodies used	The following antibodies were diluted in 1% milk in 1X TBST: anti-APOBEC3A/B/G (04A04) and anti-APOBEC3A (01D05; WB 1:1000),

Antibodies used	anti-APOBEC3B (Abcam; ab184990; WB 1:500), anti-REV1 (Santa Cruz; sc-393022, WB 1:1000), anti-SMUG1 (Abcam; ab192240; WB 1:1,000 and Santa Cruz; sc-514343; WB 1:1,000), anti-UNG (abcam; ab109214; WB 1:1,000), anti-GFP (Santa Cruz; sc-9996; WB 1:1,000), anti-β-actin (Abcam; ab8224; WB 1:3,000), anti-β-actin (Abcam, ab8227; WB 1:3,000); anti-Mouse IgG HRP (Thermo Fisher Scientific; 31432; 1:10,000), anti-Rabbit IgG HRP (SouthernBiotech; 6441-05; 1:10,000)
Validation	All the commercial antibodies used in the study showed convincing validation data presented in the relative website with knock out validated clones or positive controls (i.e. transfected cells). All the bands observed were at the expected and reported sizes, decreasing in signal if shRNAs were expressed or completely abrogated in case of KO. Custom anti-APOBEC3A/B/G (04A04) and anti-APOBEC3A (01D05) antibodies were validated against transfection controls (Extended Data Fig. 1g) and knockdown/knockout (Extended Data Fig. 1i-m; Fig. 3l).

Eukaryotic cell lines

Policy information about cell lines

Cell line source(s)	MDA-MB-453 (Cosmic Cell Line Project); BT-474 (Cosmic Cell Line Project); JSC-1 (Cosmic Cell Line Project); BC-1 (Cosmic Cell Line Project); MCF10A (ATCC); HT-1376 (ATCC); HEK293FT (ATCC)
Authentication	SNP profiling
Mycoplasma contamination	All cell lines were periodically tested for and were negative for mycoplasma contamination.
Commonly misidentified lines (See ICLAC register)	No commonly misidentified cell lines were used in this study.
